# TCGA data and patient-derived orthotopic xenografts highlight pancreatic cancer-associated angiogenesis

**DOI:** 10.18632/oncotarget.3233

**Published:** 2015-02-25

**Authors:** Jesse Gore, Kelly E. Craven, Julie L. Wilson, Gregory A. Cote, Monica Cheng, Hai V. Nguyen, Harvey M. Cramer, Stuart Sherman, Murray Korc

**Affiliations:** ^1^ Department of Medicine, Indiana University School of Medicine, Indianapolis, IN 46202, USA; ^2^ Department of Biochemistry and Molecular Biology, Indiana University School of Medicine, Indianapolis, IN 46202, USA; ^3^ Department of Surgery, Indiana University School of Medicine, Indianapolis, IN 46202, USA; ^4^ Department of Pathology and Laboratory Medicine, Indiana University School of Medicine, Indianapolis, IN 46202, USA; ^5^ The Melvin and Bren Simon Cancer Center, and the Center for Pancreatic Cancer Research, Indianapolis, IN 46202, USA; ^6^ Department of Medicine, Medical University of South Carolina, Charleston, SC 29425, USA

**Keywords:** Pancreatic cancer, Angiogenesis, TGF-β, STAT3, mouse model

## Abstract

Pancreatic ductal adenocarcinomas (PDACs) overexpress pro-angiogenic factors but are not viewed as vascular. Using data from The Cancer Genome Atlas we demonstrate that a subset of PDACs exhibits a strong pro-angiogenic signature that includes 37 genes, such as HDAC9, that are overexpressed in PDAC arising in KRC mice, which express mutated Kras and lack RB. Moreover, patient-derived orthotopic xenografts can exhibit tumor angiogenesis, whereas conditioned media (CM) from KRC-derived pancreatic cancer cells (PCCs) enhance endothelial cell (EC) growth and migration, and activate canonical TGF-β signaling and STAT3. Inhibition of the type I TGF-β receptor with SB505124 does not alter endothelial activation *in vitro*, but decreases pro-angiogenic gene expression and suppresses angiogenesis *in vivo*. Conversely, STAT3 silencing or JAK1–2 inhibition with ruxolitinib blocks CM-enhanced EC proliferation. STAT3 disruption also suppresses endothelial HDAC9 and blocks CM-induced HDAC9 expression, whereas HDAC9 re-expression restores CM-enhanced endothelial proliferation. Moreover, ruxolitinib blocks mitogenic EC/PCC cross-talk, and suppresses endothelial p-STAT3 and HDAC9, and PDAC progression and angiogenesis *in vivo*, while markedly prolonging survival of KRC mice. Thus, targeting JAK1–2 with ruxolitinib blocks a final pathway that is common to multiple pro-angiogenic factors, suppresses EC-mediated PCC proliferation, and may be useful in PDACs with a strong pro-angiogenic signature.

## INTRODUCTION

Pancreatic ductal adenocarcinoma (PDAC) is the fourth leading cause of cancer-related deaths in the United States, with dismal overall 5-year survival rates of 6% [1]. PDAC most often presents at an advanced stage and with metastatic disease, which precludes resection, and is often associated with marked chemoresistance and intense desmoplasia that may interfere with tumor blood flow and hinder drug penetration into the pancreatic tumor mass [2–5]. Nonetheless, PDAC often exhibits foci of endothelial cell (EC) proliferation, and several [6–8], but not all [9] studies, have reported a positive correlation between blood vessel density, tumor vascular endothelial growth factor-A (VEGF-A) levels, and disease progression in PDAC. Moreover, pancreatic cancer cell lines secrete biologically active VEGF-A [10], and its expression in the pancreatic cancer cells (PCCs) may be associated with enhanced local tumor spread, increased incidence of liver metastasis, and decreased patient survival [6–8].

Studies using subcutaneous and orthotopic mouse models of PDAC have suggested that anti-angiogenic therapy is effective at suppressing pancreatic tumor growth. Thus, the anti-angiogenic agent TNP-470 reduced neoangiogenesis in tumors formed by MIA-PaCa-2, ASPC-1, and CAPAN-1 PCCs, resulting in decreased tumor growth and metastatic spread [11]; targeting VEGF-A expression with a VEGF antisense construct markedly attenuated tumorigenicity in nude mice [10]; VEGF-A fused to diphtheria toxin (DT-VEGF) internalizes in target cells via VEGFRs, inhibits protein synthesis, and directly suppresses the growth of HUVEC ECs, while decreasing tumor volume, tumor spread, and microvessel density in tumors formed by HPAF-2 and ASPC-1 PCCs [12]; adenoviral vectors carrying sequences encoding soluble VEGFR-1 and VEGFR-2 [13, 14], or the VEGFR tyrosine kinase inhibitor PTK 787 [15], inhibit the growth and metastasis of pancreatic tumors in severe combined immune deficient (SCID) mice and athymic mice.

Studies with the KPC genetically engineered mouse model (GEMM) of PDAC, which expresses mutated Kras and p53 alleles in the pancreas due to Pdx1-driven Cre recombination [16], have suggested that pancreatic tumor masses have a paucity of blood flow [17–19] that can be enhanced to facilitate drug delivery to the tumor mass using therapies that promote tumor angiogenesis [19], or by targeting the stroma [17, 18]. However, a separate GEMM-based study using KP^fl/+^C mice, which also express mutated Kras but have p53 haploinsufficiency, suggested that stroma depletion worsens disease, but that it is reversible by anti-angiogenesis therapy with a VEGFR2 blocking antibody [20, 21]. Thus, whether angiogenesis should be targeted in PDAC is not entirely clear.

Although studies in certain murine models raise the possibility that VEGFR signaling may have an important role in PDAC, targeting VEGF-A either with bevacizumab, an anti-VEGF-A antibody, or with VEGF Trap, which sequesters VEGF, has failed in clinical trials in PDAC patients [22, 23]. This therapeutic failure might be due to the fact that PDAC also overexpresses additional angiogenic factors, such as TGF-β, hepatocyte growth factor (HGF), fibroblast growth factors (FGFs), platelet derived growth factor (PDGF), and pro-angiogenic cytokines [24, 25]. Nonetheless, a recent Phase II clinical trial indicated that vatalanib, an inhibitor of VEGF and PDGF receptors, slightly improved survival in metastatic PDAC [26]. Therefore, the role of angiogenesis in PDAC is more complex than previously appreciated and targeting multiple-angiogenic pathways may be more effective than targeting a single pathway.

We recently established a GEMM of PDAC [27] in which mice that express RB with LoxP sites in the introns flanking exon 19 were bred with KC mice (Kras-Cre recombinase). KC mice carry an oncogenic *Kras* (*Kras^G12D^*) allele within its own locus downstream of its endogenous promoter and silenced by a LoxP-Stop-LoxP element (LSL) upstream of the transcriptional start site [28, 29]. The KRC compound mutant mice express oncogenic Kras in the pancreas which is devoid of RB due to Pdx1-driven Cre recombination (22). KRC mice develop pancreatic intraepithelial neoplasia (PanIN) -1 and -2 lesions at 2 weeks after birth and exhibit rapid PanIN progression to murine PDAC (mPDAC) (22) with increased expression of several cytokines, such as transforming growth factor betas (TGF-βs) and TNF-α, which are also overexpressed in human PDAC and are pro-angiogenic [30, 31]. Moreover, PDAC in humans is often associated with loss of RB function [32], underscoring the potential relevance of the KRC GEMM to the human disease.

In the present study, using TCGA data, we determine that ~12% of PDACs exhibits a pro-angiogenic gene signature. We also establish a novel patient-derived orthotopic xenograft (PDOX) model directly from samples obtained by fine needle aspiration (FNA) during endoscopic ultrasonography (EUS) and demonstrate that ECs are present in the original tumor and that these human ECs survive when implanted into immunodeficient mice. Moreover, we show that KRC mPDACs express a pro-angiogenic gene signature that overlaps with many of the genes in the above TCGA subset, and that inhibiting JAK1–2 signaling markedly prolongs survival in this model and suppresses cancer progression *in vivo*, while preventing ECs from stimulating PCC growth in culture. Therefore, targeting JAK1–2 signaling may be especially useful in the subset of PDAC patients that exhibit a pro-angiogenic gene signature.

## RESULTS

### A subset of PDAC patients express a strong angiogenesis gene profile and patient-derived orthotopic xenografts exhibit tumor angiogenesis

**Figure 1:**
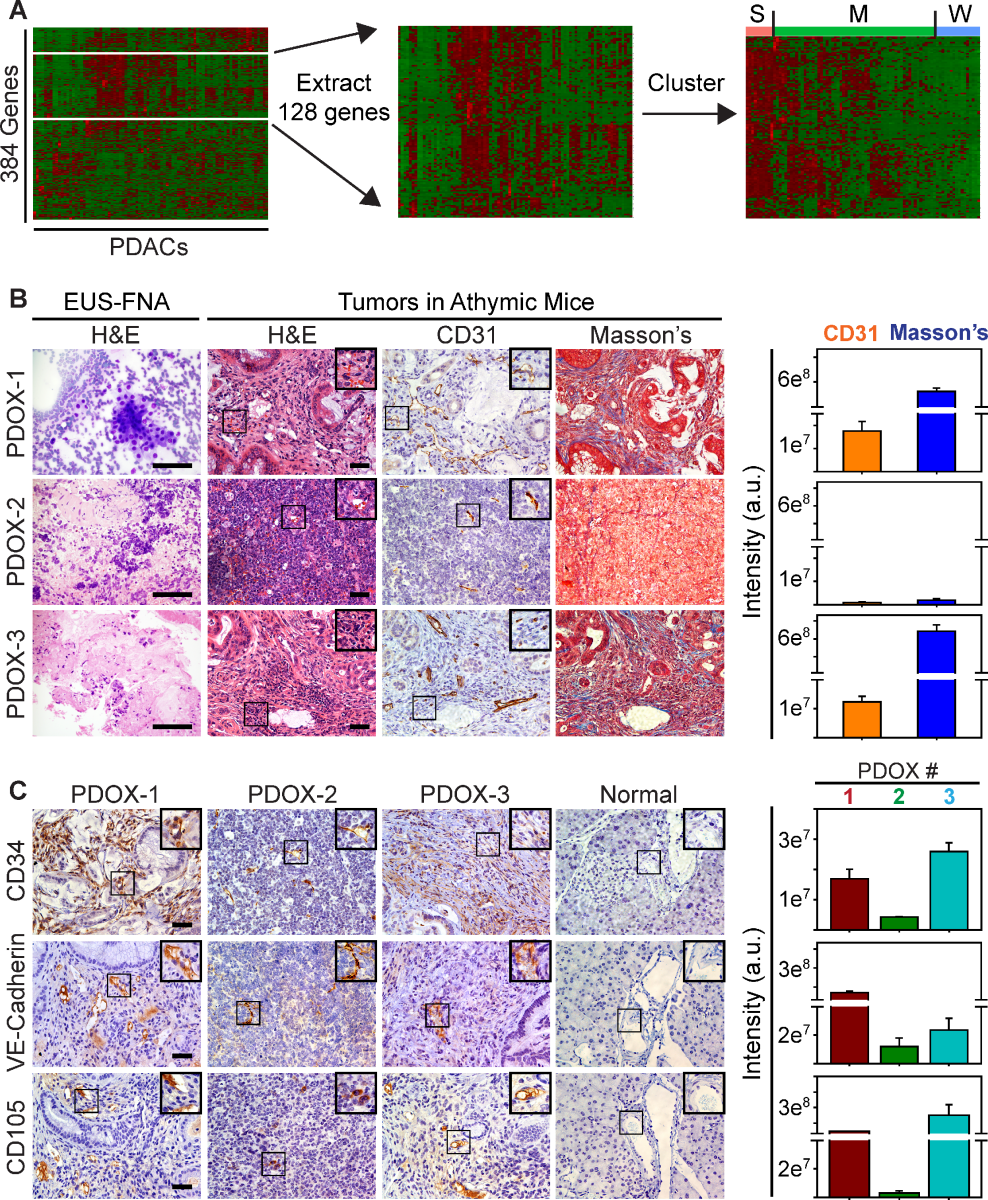
A Subset of Human PDACs exhibit a strong angiogenic gene signature **(A)** Hierarchical clustering of TCGA data show that genes annotated to angiogenesis (white lines) are up-regulated in some human PDACs (left). Extraction (middle) and re-clustering (right) shows that many of these angiogenesis genes are up-regulated in a subset of PDACs (S = strong), whereas some (M = moderate) or few of these genes (W = weak) are increased in other PDACs. **(B)** H&E staining shows cytology of EUS-FNA samples (left) and histology of EUS-PDOX tumors in athymic mice. CD31 immunohistochemistry shows that EUS-PDOX tumors harbor ECs in the collagen-rich stroma highlighted by Masson's Trichrome staining of serial sections. **(C)** Human-specific CD34 (top), VE-Cadherin (middle) and CD105 (bottom) antibodies react with ECs in EUS-PDOX tumors, but fail to react with ECs in normal murine pancreata. Quantitation (right) of CD31 and Masson's Trichrome (B), or CD34 (upper), VE-Cadherin (middle) and CD105 (lower panel) (C) pixel intensity shows that stroma content and the abundance of ECs in PDOX2 are decreased compared with PDOX1 and PDOX3. Shown in (B–C) are representative images from three EUS-PDOX tumors. Insets show magnified images of boxed areas. Scale bars, 50 μm.

To assess the angiogenic potential of human PDACs we analyzed an RNASeq dataset from 85 PDACs in The Cancer Genome Atlas (TCGA), focusing on genes annotated to angiogenesis Gene Ontology (GO) terms. Hierarchical clustering revealed that out of 384 genes annotated to angiogenesis, approximately 128 were up-regulated in some tumors (Figure 1A). We therefore extracted this gene set, and performed a second cluster analysis to determine whether any patients exhibited similar angiogenesis gene expression profiles (Figure 1A). Based on this analysis, 10/85 (~12%) PDAC patients were identified that harbored tumors in which multiple angiogenesis genes were up-regulated, suggesting that they exhibit a strong angiogenesis gene signature (Figure 1A). By contrast, 59/85 PDACs (~69%) exhibited variable expression levels of these genes, suggesting that they have a moderate angiogenesis signature, whereas 16/85 PDACs (~19%) displayed a weak signature in which few genes were elevated (Figure 1A). Differential expression analysis of tumors exhibiting a strong signature with those exhibiting a weak signature revealed that 77 angiogenesis genes were significantly up-regulated, 63 of which were pro-angiogenic (Supplementary Table 1).

To determine if human PDACs could promote tumor angiogenesis in mice, we established a new model in which EUS-FNAs were directly implanted into the pancreata of athymic mice. Seven tissue samples were obtained by EUS-FNA, and all seven patients had suspected PDAC at biopsy, which was confirmed by cytology (Figure 1B). Of these, 5 formed large intrapancreatic tumors (Supplementary Figure 1A) that were palpable within 2 months, and the mean time to sacrifice was 5.7 ± 1.6 months. These mice were termed EUS-PDOX mice to indicate that they are patient-derived orthotopic xenografts generated with EUS-FNAs. Two EUS-PDOX tumors were established from EUS-FNAs of resectable tumors, and their tumor histopathology was consistent with that of the resected tumor, including the formation of mucinous ascites from a mucin-producing adenocarcinoma (EUS-PDOX1; Supplementary Figure 2A). By contrast, the other three EUS-PDOX tumors were established from EUS-FNAs of advanced stage tumors that were unresectable (Supplementary Figure 2B).

To evaluate these tumors for angiogenesis we used the CD31 endothelial marker. CD31-positive cells were present in the stroma of all EUS-PDOX tumors (Figure 1B) and in adjacent normal pancreas (Supplementary Figure 1B). To determine if endothelial cells (ECs) within the tumor mass were host- (murine) or biopsy- (human) derived, we used a human-specific CD34 antibody that did not mark ECs in normal murine pancreata (Figure 1C), or in orthotopic tumors generated by intrapancreatic injection of human PCCs into athymic mice (Supplementary Figure 1C). Consistent with the localization of CD31-positive cells, CD34 immunoreactivity was present in the stromal compartment of all EUS-PDOX tumors (Figure 1C). Moreover, human-specific VE-Cadherin and CD105 (endoglin) antibodies marked ECs in EUS-PDOX tumors (Figure 1C), but were non-reactive with murine ECs in normal pancreata (Figure 1C) or in human PCC-generated orthotopic tumors (Supplementary Figure 1C). Thus, the tumor endothelium in first (F0) EUS-PDOX tumors that formed was, in part, patient-derived. EUS-PDOX tumors were either moderately differentiated with abundant stroma, or poorly differentiated with a paucity of stroma (Figure 1B–1C, Supplementary Figure 2), and these histological features were maintained after their first *in vivo* passage (F1; Supplementary Figure 1D). Although F1 tumors continued to harbor ECs, these ECs no longer expressed any human markers (Supplementary Figure 1D), indicating that as these tumors developed and grew they were able to readily recruit host-derived ECs.

**Figure 2:**
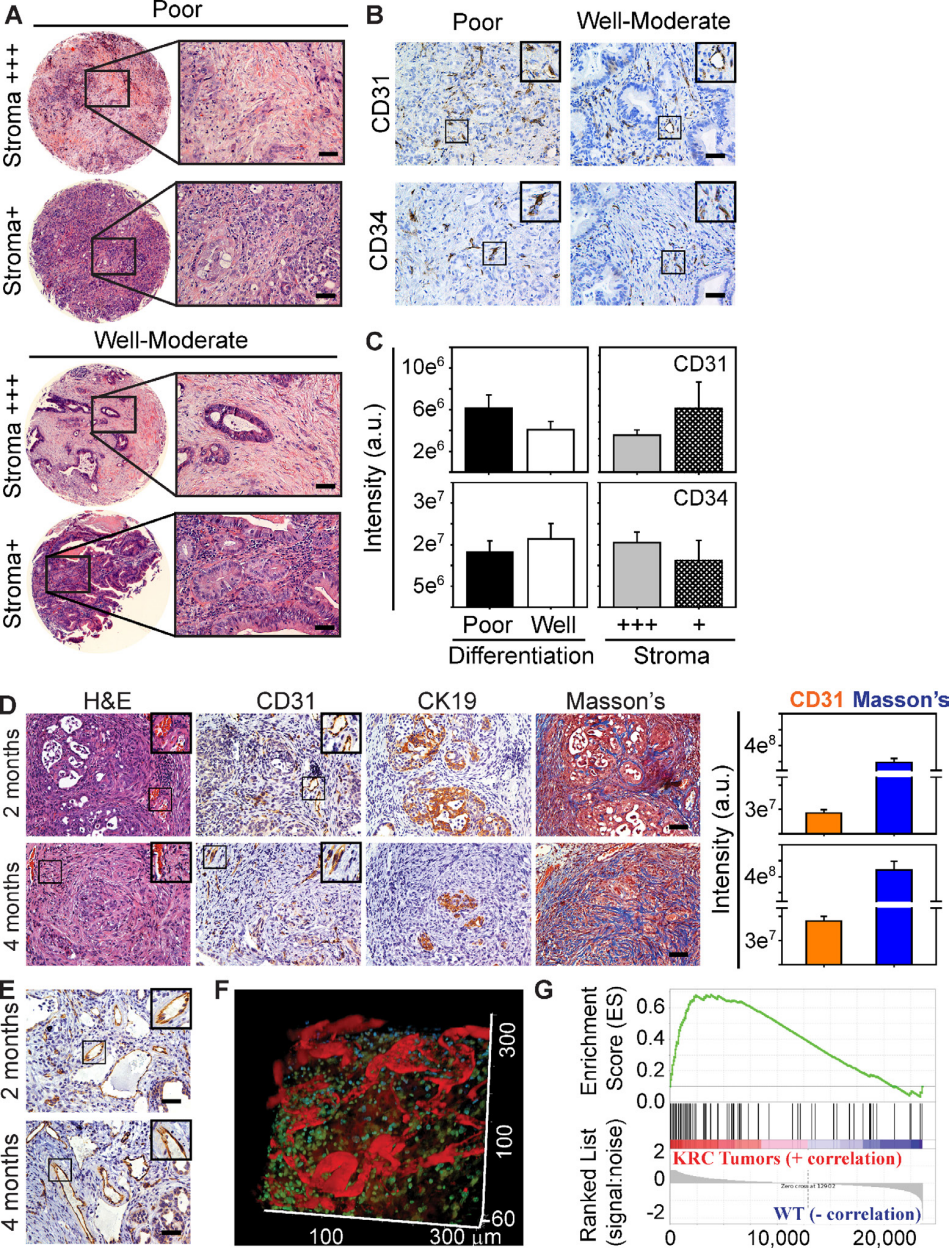
Subsets of Human PDACs and KRC murine PDACs exhibit angiogenesis **(A)** H&E staining shows that tumors in a human PDAC tissue microarray exhibit poor or well-to-moderate differentiation, and that some have abundant stroma (stroma+++), whereas others are relatively stroma-deficient (stroma+). **(B)** ECs are present in poor and well-moderately differentiated PDACs as evidenced by the presence of CD31 (top) and CD34 (lower) immunoreactivity. Insets show magnified images of boxed areas. Shown in (A–B) are 4/54 human PDACs. **(C)** Pixel intensity quantitation shows CD31 (top) and CD34 (bottom) immunoreactivity is similar when comparing poor (closed bars) with well-moderate (open bars) PDACs, or when comparing stroma+++ (gray bars) with stroma+ (hatched bars) PDACs. Data are presented as mean ± SEM. **(D)** KRC mPDACs harbor ECs adjacent to CK19-positive cancer cells in the collagen-rich stroma as evidenced by Masson's Trichrome staining of serial sections. Quantitation (right) of CD31 and Masson's Trichrome staining shows that EC abundance and stromal content increases from postnatal months 2 to 4. **(E)** CD31-positive vessels are also present throughout KRC mPDACs. Shown in (D–E) are representative images from 5 KRC mice at each age. Insets in (B, D–E) are magnified images of boxed areas. Scale bars, 50 μm. **(F)** Intravital microscopy shows that KRC tumors have blood flow as evidenced by the abundance of dextran-positive (red) vessels. Shown is a representative image from 1 of 2 KRC mice. **(G)** Gene set enrichment analysis (GSEA) comparing KRC tumor array data with TCGA data shows that genes up-regulated in KRC tumors correlate with genes up-regulated in the strong pro-angiogenic gene signature subgroup (family-wise error rate (FWER) < 0.001).

Quantitation of endothelial and stromal markers in EUS-PDOX tumors revealed that tumors with abundant stroma, rich in collagens as determined by Masson's trichrome (Figure 1B) and Picosirius red (Supplementary Figure 1E), harbored more ECs than stroma-deficient tumors (Figure 1B–1C). It has been recently proposed that stroma-depleted mPDACs are poorly differentiated, but associated with either increased [20] or decreased [33] angiogenesis. To clarify this issue and determine whether angiogenesis is dictated by the differentiation status and extent of desmoplasia in PDAC, we used a human PDAC tissue microarray (TMA) in which 26 tumors were poorly differentiated and 28 were well-to-moderately differentiated (Figure 2A). Irrespective of tumor differentiation, 45/54 tumors were rich in stroma (stroma+++), whereas 9 were relatively stroma-deficient (stroma+), allowing us to determine if angiogenesis correlated with stroma abundance. CD31 or CD34 immunoreactivity was present in all PDACs (Figure 2B), and was strong in 15/54 tumors, but moderate in 20/54 and weak in 19/54 (Supplementary Figure 3), suggesting that some PDACs exhibit robust angiogenesis. However, when comparing poorly differentiated with well-to-moderately differentiated tumors, or stroma-rich vs stroma-poor tumors, there were no differences in either CD31 or CD34 immunoreactivity between these groups (Figure 2C). Therefore, in humans, PDAC angiogenesis is not necessarily associated with poorly-differentiated or stroma-deficient tumors.

### KRC mice exhibit abundant tumor angiogenesis and a pro-angiogenic gene signature

KRC mice, which express oncogenic *Kras* in the pancreas, but lack RB function exhibit rapid PanIN formation and progression to murine PDAC (mPDAC) with a high frequency [27]. Akin to human PDAC, which is commonly associated with a high frequency of *KRAS* mutations (95%) and loss of RB function [32], KRC mPDACs express high levels of pro-angiogenic cytokines [27]. Therefore, we next sought to determine whether KRC mPDACs exhibit angiogenesis. As in EUS-PDOX tumors, CD31 immunoreactivity in KRC mPDAC was present in sinusoidal-like blood vessels within the collagen-rich stroma adjacent to CK19-positive cancer cells (Figure 2D), and in relatively larger blood vessels within the stromal compartment (Figure 2E). Moreover, intravenous injection of TRITC-conjugated dextran followed by intravital imaging using two-photon confocal microscopy, demonstrated many dextran-positive vessels (Figure 2F), confirming the presence of blood flow.

We next conducted an array analysis using KRC tumor-derived RNA to determine if they exhibit a pro-angiogenic gene expression profile. Gene Ontology (GO) analysis revealed that KRC tumors exhibited significant enrichment of pro-angiogenic processes (Supplementary Figure 4A). Moreover, gene set enrichment analysis (GSEA) indicated that these genes were often the same as those up-regulated in human PDACs with a strong angiogenesis gene signature (Figure 2G). Thus, compared with the 77 gene TCGA signature, 42 genes were differentially expressed in KRC tumors, 37 of which were pro-angiogenic (Supplementary Table 2). Together, these data suggest that like human PDAC, KRC mPDACs exhibit a robust pro-angiogenesis signature.

### A pro-angiogenic gene signature is present in KRC PCCs

Increased expression of pro-angiogenic genes in KRC tumors could arise as a consequence of their up-regulation in the cancer cells. Therefore, we next evaluated KRC-derived PCCs for a pro-angiogenic gene expression profile. Accordingly, we conducted a GO analysis of microarray data comparing KRC PCCs with PCCs derived from KC tumors, which also express oncogenic *Kras*, but retain RB function and express low levels of pro-angiogenic cytokines [32]. KRC PCCs exhibited significant enrichment of pro-angiogenic processes (Supplementary Figure 4B), and increased expression of 47 pro-angiogenic genes (Supplementary Table 3). By contrast, only 8 anti-angiogenic genes were differentially expressed, 4 of which were down-regulated (Supplementary Table 3). qPCR validated the arrays and confirmed that KRC tumors and PCCs expressed relatively high levels of *Ctgf, Cyr61, Egfr, Nrp2, Serpine1, Tgbr1* and *Vegfc* mRNA (Supplementary Figure 4C–4D). By contrast, *Vegfa* mRNA levels were similar in KRC and KC cells, in agreement with the observation that oncogenic Kras, *per se*, can up-regulate *Vegfa* mRNA expression [34]. Thus, KRC tumors and PCCs exhibit increased expression of multiple pro-angiogenic factors which are reflective of the gene expression profile seen in human PDAC.

### TGF-β promotes angiogenesis indirectly

**Figure 3:**
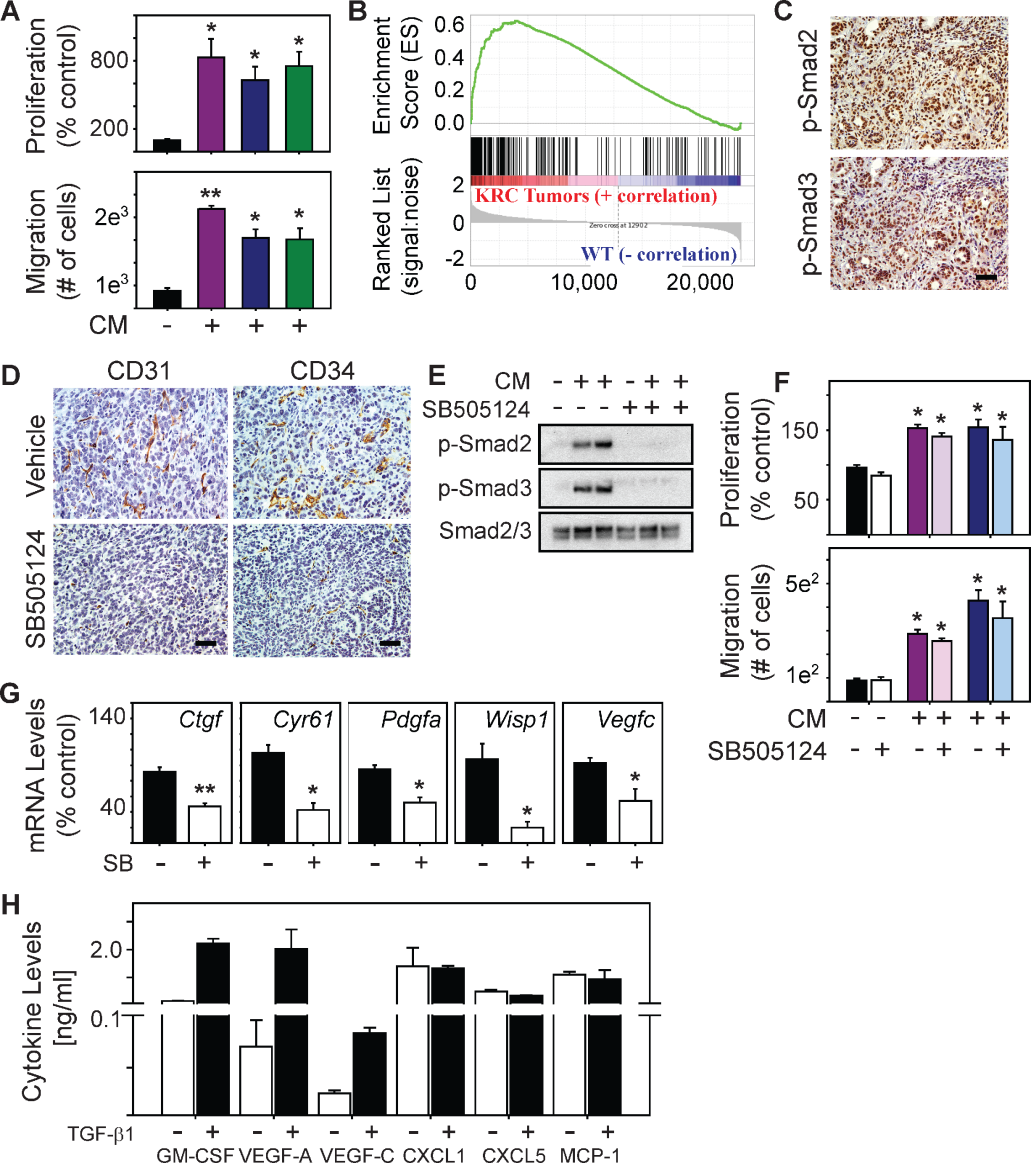
TβRI inhibition does not block endothelial activation but suppresses angiogenic gene expression in PCCs **(A)** Compared with control media (closed bars), conditioned media (CM) from three KRC cell lines (purple, green or blue bars) significantly enhance EC proliferation (top) and migration (bottom). **(B)** GSEA shows that genes up-regulated in KRC tumors correlate with genes up-regulated by TGF-β (FWER < 0.001). **(C)** Canonical TGF-β signaling pathways are active in KRC tumors as evidenced by the abundance of nuclear p-Smad2 (top) and p-Smad3 (bottom). **(D)** Vehicle-treated tumors (top) display abundant CD31 (left) and CD34 (right) immunoreactivity, both of which are markedly attenuated in SB505124-treated tumors. Shown in (C–D) are representative images from 3 mice per group. Scale bars, 50 μm. **(E)** CM from KRC cells markedly increase p-Smad2 and p-Smad3 levels in ECs, which is blocked by SB505124 [2 μM]. Shown are representative immunoblots from three independent experiments. **(F)** SB505124 [2 μM] does not prevent KRC CM from enhancing EC proliferation (top) or migration (bottom). Data in (A, F–H) are mean ± SEM. **P < 0.01; *P < 0.05. **(G)** SB505124 (2 μM] significantly attenuates the levels of the indicated mRNAs in KRC PCCs. **(H)** ELISA shows the levels of the indicated cytokines in KRC CM in the absence (open bars) or presence (closed bars) of TGF-β1. Data are mean ± SD from two different cell lines.

We next assessed the ability of conditioned media (CM) from KRC cells to enhance the proliferation and migration of murine SVEC4–10 ECs that are commonly used to study angiogenesis pathways *in vitro* [35]. CM from three KRC-derived PCCs markedly enhanced EC proliferation and migration (Figure 3A), suggesting that KRC cells may secrete factors that promote EC proliferation *in vivo*. To identify mechanisms through which KRC cell-derived factors could promote angiogenesis *in vivo*, we conducted an Ingenuity Pathway Analysis (IPA) of our KRC tumor array data. Based on the overall gene expression profile of KRC tumors, IPA identified TGF-β as the top regulator of gene expression (*P* = 1.91 × 10^−16^). GSEA confirmed these findings, and indicated that genes up-regulated in KRC tumors correlated strongly with genes up-regulated by TGF-β (Figure 3B). Moreover, KRC mPDACs (Figure 3C) and human PDACs [32] are often associated with nuclear p-Smad2 and p-Smad3, indicating that canonical TGF-β signaling pathways are active in both settings, and raising the possibility that PDAC-derived TGF-βs could promote angiogenesis [36].

KRC PCCs also exhibit a gene expression profile that reflects active TGF-β signaling [32]. Moreover, in a syngeneic orthotopic model using KRC cells, inhibition of TGF-β attenuates tumor growth and metastasis, and markedly prolongs survival [32]. Inasmuch as blocking TGF-β signaling suppresses tumor angiogenesis in immune-deficient orthotopic models using human PCCs [37], we next evaluated the consequences of TGF-β type I receptor (TβRI) kinase inhibition with SB505124 on angiogenesis in a syngeneic KRC orthotopic model. Compared with vehicle-treated tumors, CD31 and CD34 immunoreactivity was attenuated in the tumors of mice treated with SB505124 a (Figure 3D), suggesting that TGF-β signaling enhances angiogenesis in these tumors *in vivo*. These pro-angiogenic actions could be due to direct effects on ECs, or through the up-regulation of pro-angiogenic genes in the PCCs and cells within the tumor microenvironment (TME). To explore whether TGF-β acted directly on ECs, we next assessed the ability of CM derived from KRC PCCs to activate canonical TGF-β signaling in SVEC4–10 ECs. Indeed, KRC-derived CM increased Smad2 and Smad3 phosphorylation in ECs, which was blocked by SB505124 (Figure 3E), pointing to TGF-β pathway activation in the ECs. However, SB505124 failed to block the ability of KRC-derived CM to stimulate EC proliferation or migration (Figure 5F), indicating that TGF-β does not directly enhance angiogenesis.

### STAT3 is active in tumor endothelial cells

**Figure 4:**
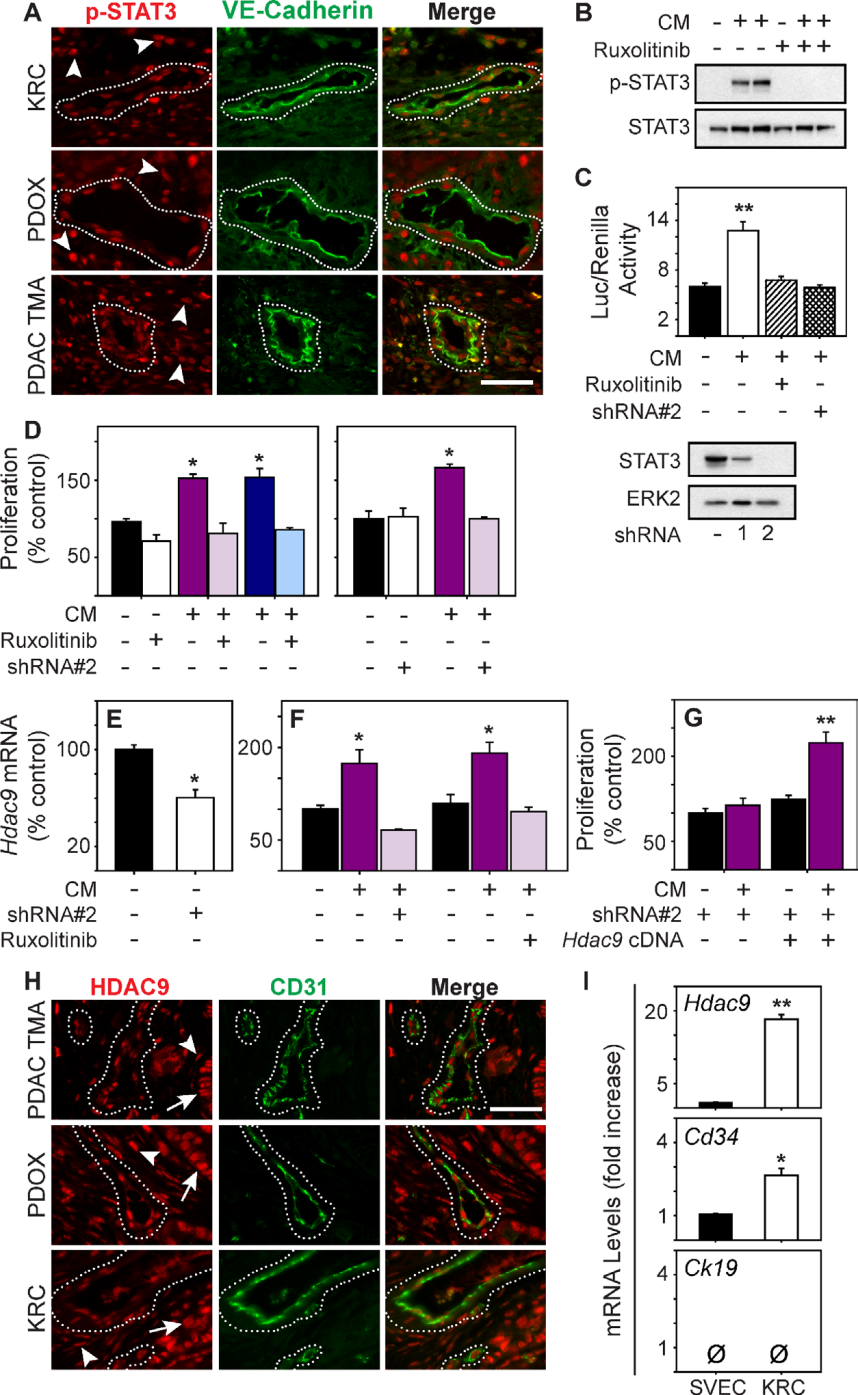
STAT3 is active in PDAC tumor endothelia and enhances HDAC9 expression to promote endothelial proliferation **(A)** p-STAT3 (red) is abundant in the nuclei of VE-Cadherin-positive vessels (green, outlined) and surrounding stromal cells (arrowheads) in KRC (top), EUS-PDOX tumors (middle), and human PDACs (bottom). **(B)** KRC CM markedly increases p-STAT3 levels in ECs, which is blocked by ruxolitinib [100 nM]. **(C)** KRC CM significantly enhances STAT3 luciferase reporter activity in ECs (top), which is blocked by ruxolitinib [100 nM] or a STAT3-targeting shRNA (shRNA#2). Immunoblotting (lower panel) shows the knockdown efficiency of STAT3-targeting shRNAs. ERK2 confirms equivalent lane loading. Shown in (B–C) are representative immunoblots from three independent experiments. **(D)** CM from KRC cells significantly enhances EC proliferation, but in the presence of ruxolitinib ([100 nM], left) or in ECs transduced with a STAT3-targeting shRNA#2 (right) CM fails to enhance EC proliferation. **(E)** *Hdac9* mRNA levels are significantly decreased in ECs transduced with STAT3-targeting shRNA#2 (open bar). **(F)** CM from KRC cells significantly increases *Hdac9* mRNA levels in ECs, but in the presence of shRNA#2 or ruxolitinib [100 nM] CM fails to up-regulate *Hdac9*. **(G)** CM fails to stimulate the proliferation of ECs transduced with shRNA#2, but when these ECs are transfected with an *Hdac9* cDNA construct, CM significantly enhances EC proliferation. **(H)** HDAC9 (red) is abundant in the nuclei of CD31-positive vessels (green, outlined) and in surrounding stromal (arrowheads) and cancer cells (arrows) in EUS-PDOX (middle) and KRC PDACs (bottom) as evidenced by co-localization with DAPI (blue) in CD31-cadherin-positive vessels (outlined). **(I)** Compared with SVEC4–10 ECs, *Hdac9* and *Cd34* are significantly increase in KRC tumor-derived ECs, whereas *Ck19* is absent in both. Shown in (A) and (H) are representative images from three KRC or EUS-PDOX tumors, or the TMA. Scale bars, 50 μm. Data in (C–G, I) are mean ± SEM. *P < 0.05, and **P < 0.01.

Analysis of KRC PCC array data revealed that 34 of the 47 pro-angiogenic genes up-regulated in KRC cells were predicted to be TGF-β targets (Supplementary Table 3). To determine if TGF-βs promote pro-angiogenic gene expression in KRC cells, we suppressed TβRI signaling with SB505124, and assayed *Ctgf* and *Wisp1*, which were elevated in the human PDACs with a pro-angiogenic signature, and *Cyr61, Pdgfa* and *Vegfc*, which are known to promote angiogenesis and which have also been reported to be expressed at high levels in human PDAC [25, 38, 39]. SB505124 markedly suppressed the levels of all five mRNAs (Figure 3G). Moreover, out of 25 cytokines assayed by multiplex ELISA, only CXCL1, CXCL5, MCP-1, GM-CSF, VEGF-A and VEGF-C were readily detected in KRC CM, and TGF-β1 increased the levels of the latter three (Figure 3H, Supplementary Table 4). Importantly, TGF-β increased GM-CSF by 14.7-fold and VEGF-A by 42-fold, whereas IL6, a potent inducer of STAT3, was below the level of detection (Supplementary Table 4). All six of the detectable factors are pro-angiogenic and activate STAT3, an oncogene and survival factor that can be activated by many additional cytokines and growth factors [40, 41], that has been implicated in promoting tumor angiogenesis [42] and modulating the TME [43]. Therefore, we next sought to determine whether STAT3 is active in the endothelium of KRC, EUS-PDOX and human tumors. Indeed, phosphorylated STAT3 (p-STAT3) was not only abundant in the nuclei of PCCs and cancer-associated fibroblasts (CAFs) in KRC tumors, but was also frequently present in ECs, identified using a murine-specific VE-Cadherin antibody (Figure 4A). Similarly, a human-specific VE-Cadherin antibody revealed that nuclear p-STAT3 was present in ECs in EUS-PDOX tumors, and in all VE-Cadherin-positive vessels seen in 32/54 (59%) human PDAC tissues (Figure 4A). Thus, STAT3 activation is common in PDAC endothelia.

**Figure 5:**
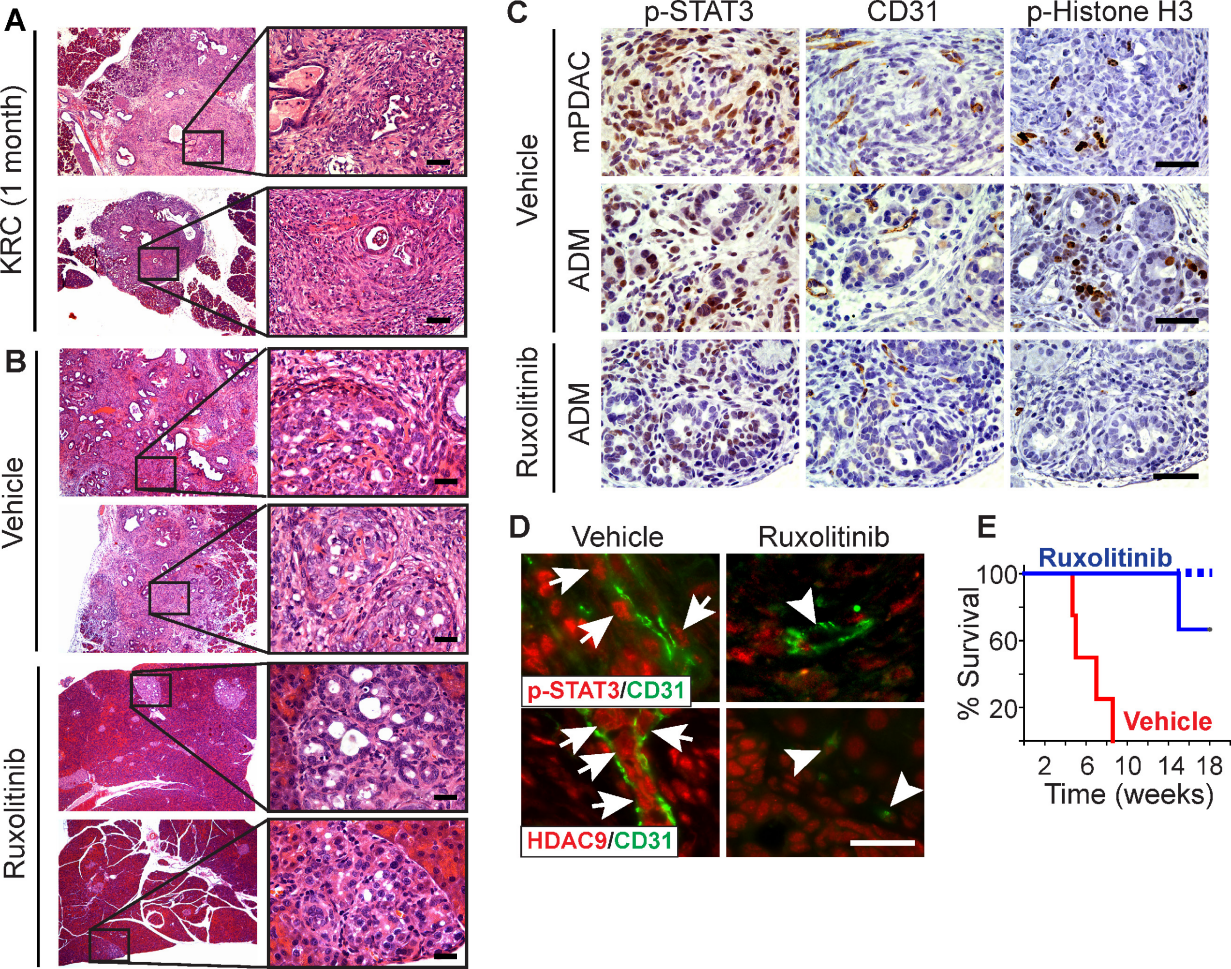
Ruxolitinib suppresses mPDAC progression and prolongs survival of KRC mice **(A–B)** H&Es show that KRC pancreata often exhibit ADM, PanIN and mPDAC at postnatal month 1 (A), and that vehicle-treated mice display abundant lesions and mPDAC, whereas ruxolitinib-treated pancreata are mostly normal and only display small foci of ADM (B) Shown are representative images from two mice per group. Right panels are high magnification images of boxed areas. **(C)** Nuclear p-STAT3 (left) is abundant in KRC mPDACs (top) and ADM (middle) in vehicle-treated mice, whereas ADM in ruxolitinib-treated mice (bottom) have weak p-STAT3 immunoreactivity. mPDACs and ADM in vehicle-treated mice also have abundant ECs and are highly proliferative as evidenced by the presence CD31 and p-Histone H3 immunoreactivity, respectively. ADM in ruxolitinib-treated mice have few CD31-positive ECs, and p-Histone H3 is mostly absent. **(D)** VE-cadherin-positive ECs (green) in vehicle-treated KRC mice harbor nuclear p-STAT3 (top panels, red, arrows), whereas ECs in ruxolitinib-treated mice lack nuclear p-STAT3 (arrowheads). CD31-positive ECs vehicle-treated KRC mice also exhibit strong, nuclear HDAC9 immunoreactivity (bottom panel, red, arrows) that is markedly attenuated in ECs in ruxolitinib-treated mice (arrowheads). All images were acquired using the same exposure time. Scale bars in (A–D), 50 μm. **(E)** Kaplan-Meier analysis shows that compared to vehicle (red line), ruxolitinib (blue line) significantly (*P* = 0.018) prolongs survival of KRC mice. Dashed line indicates that 2 ruxolitinib-treated mice were alive beyond postnatal week 18.

### STAT3 is required for endothelial cell activation by PCCs

We next determined if PCC-derived factors activate endothelial STAT3 by using CM from KRC PCCs. CM robustly increased STAT3 phosphorylation and STAT3-dependent transcription in ECs (Figure 4B–4C), which was completely blocked by the JAK1–2 inhibitor, ruxolitinib, or by silencing STAT3 in ECs with a shRNA that suppressed STAT3 to undetectable levels (Figure 4C). Ruxolitinib and STAT3 silencing also blocked the ability of CM to stimulate EC proliferation (Figure 4D). To determine if these inhibitory effects were associated with impaired up-regulation of pro-angiogenic genes within ECs, we next focused on HDAC9, since it was present in both human and KRC pro-angiogenic gene signatures, and is required for EC sprouting and tube formation *in vitro* and vessel formation *in vivo* [44]. *Hdac9* mRNA levels were readily detectable in SVEC4–10 ECs, but were significantly reduced in STAT3-silenced ECs (Figure 4E). Moreover, STAT3 silencing or ruxolitinib prevented KRC CM from significantly increasing *Hdac9* mRNA levels (Figure 4F). Conversely, transfection of murine *Hdac9* cDNA into STAT3-silenced ECs restored the ability of KRC CM to stimulate the growth of these cells (Figure 4G). Together, these results suggest that KRC-derived factors promote EC growth through STAT3-dependent pathways that are required for up-regulation of pro-angiogenic genes in ECs, including STAT3-mediated upregulation of HDAC9, which stimulates EC growth

Given that STAT3 enhanced HDAC9 expression in cultured ECs, and that STAT3 is active in PDAC ECs, we next sought to determine whether HDAC9 was present in ECs in human and KRC tumors. HDAC9 was abundant in both PCC and CAF nuclei in human PDACs, and in CD31-positive blood vessels in these tissues (Figure 4H). HDAC9 was also abundant in nuclei of cancer cells, CAFs and CD31-positive vessels in EUS-PDOX tumors and KRC mPDACs (Figure 4H). Moreover, in ECs derived from KRC tumors that were devoid of the epithelial marker CK19 but express the endothelial marker CD34, *Hdac9* mRNA levels were increased 18-fold compared with the levels in SVEC4–10 ECs (Figure 4I), suggesting that HDAC9 is present at high levels PDAC ECs.

### Ruxolitinib attenuates PDAC growth and prolongs survival

Endothelial recruitment and growth is an important aspect of tumor biology, including the progression of pre-malignant lesions to cancer [45]. KRC mice develop acinar-to-ductal metaplasia (ADM) and PanIN lesions that rapidly and frequently progress to mPDAC [27]. Moreover, lesion initiation and progression occurs in conjunction with the appearance of inflammatory infiltrates and increased cytokine expression [27]. Therefore, we next used this GEMM to determine if inhibiting JAK1–2 with ruxolitinib could act to impede angiogenesis and suppress PanIN progression and mPDAC growth. Accordingly, we administered ruxolitinib to KRC mice at postnatal month 1, an age at which ADM, PanIN and mPDAC are commonly observed in the pancreas (Figure 5A). After 3 weeks of therapy, we evaluated their pancreata for extent and severity of disease. All vehicle-treated mice exhibited multiple foci of ADM, PanIN and mPDAC (Figure 5B) that occurred in conjunction with strong, nuclear p-STAT3 immunoreactivity (Figure 5C). The cancer cells and ADM were proliferative as evidenced by the presence of nuclear phosphorylated Histone H3 (p-Histone H3), and were surrounded by an abundance of CD31-positive ECs (Figure 5C). Moreover, ECs in vehicle-treated mice frequently harbored nuclear p-STAT3 and HDAC9 (Figure 5D). Remarkably, the pancreata of KRC mice receiving ruxolitinib were mostly normal, and only displayed small foci of ADM (Figure 5B) that exhibited weak p-STAT3 immunoreactivity and markedly attenuated proliferation, and were associated with few ECs (Figure 5C) in which nuclear p-STAT3 and HDAC9 immunoreactivity was markedly attenuated (Figure 5D). Moreover, in a survival study, 100% of ruxolitinib-treated mice were alive and healthy at postnatal week 14, with only one mouse succumbing at postnatal week 15 (Figure 5E). By contrast, all vehicle-treated mice succumbed by postnatal week 8.5 (Figure 5E). Therefore, ruxolitinib attenuates ADM progression to PanIN and mPDAC, while suppressing angiogenesis and markedly prolonging survival in this autochthonous model.

In contrast to KRC tumors, mPDACs arising in KPC mice which express oncogenic Kras together with mutated p53, did not exhibit *Hdac9* up-regulation or a pro-angiogenic gene signature that was present in KRC and human PDACs (Supplementary Figure 5A–5B). Therefore, we next used this GEMM to determine whether targeting JAK1–2 with ruxolitinib suppresses PanIN progression and mPDAC growth in a GEMM lacking tumor angiogenesis [46]. We administered ruxolitinib to KPC mice at postnatal month 3 when they often exhibited ADM, PanIN and mPDAC (Supplementary Figure 5C). After 3 weeks of therapy, pancreatic histology from vehicle- and ruxolitinib-treated mice were similar, and frequently exhibited abundant ADM, PanIN and mPDACs, with many p-Histone H3-positive nuclei but few CD31-positive ECs (Supplementary Figure 5D–5E). Therefore, ruxolitinib failed to suppress cancer cell proliferation or mPDAC progression in KPC mice.

**Figure 6:**
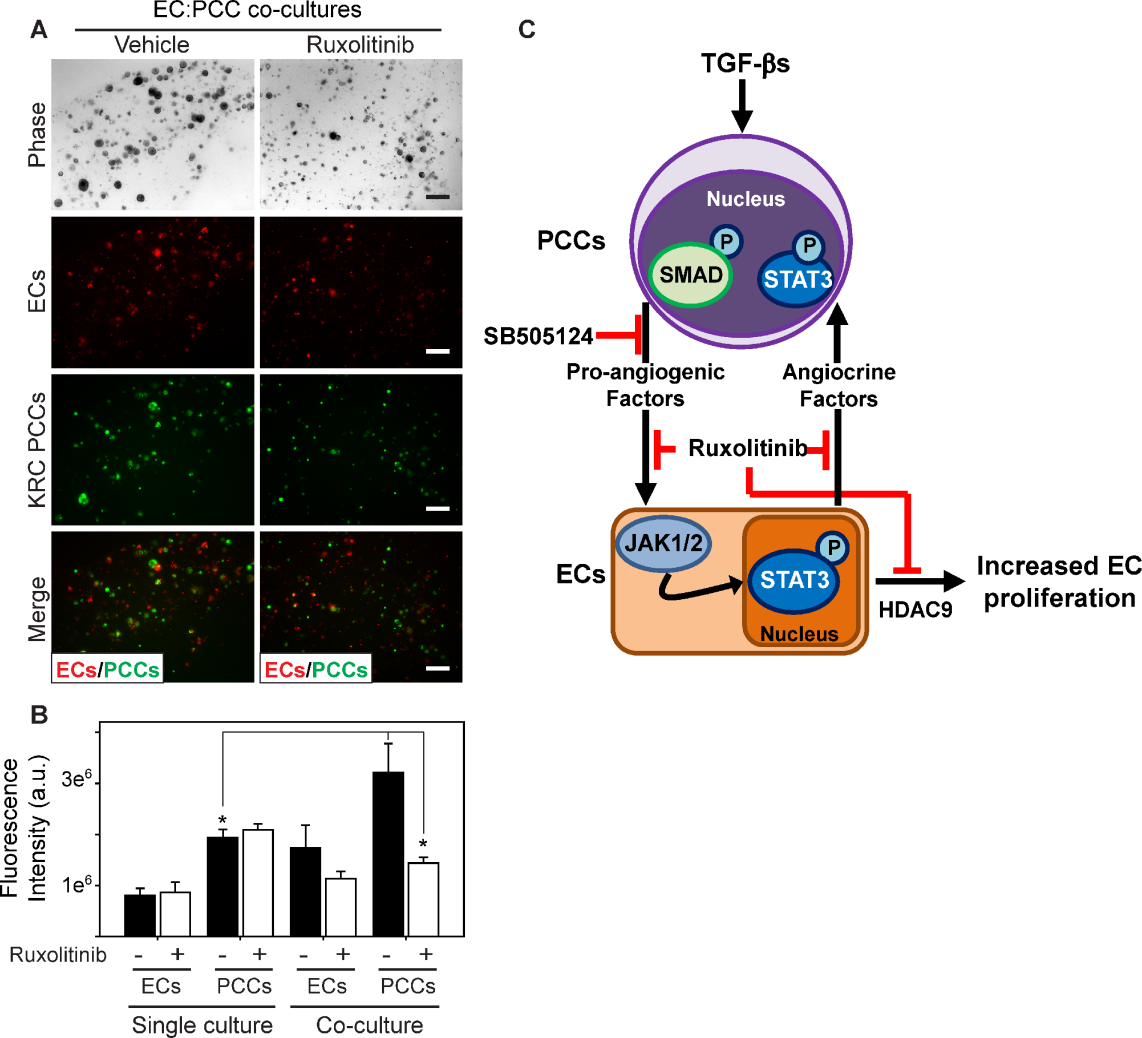
Ruxolitinib suppresses mitogenic cross-talk between endothelial cells and PCCs **(A)** 3D co-cultures of ECs (red) and KRC PCCs (green) shows that compared with vehicle (DMSO, left), ruxolitinib ([100 nM], right) suppresses PCC growth. Shown are representative phase contrast and fluorescent images taken on day 8. Scale bars, 200 μm. **(B)** Fluorescence intensity quantitation shows that compared with 3D cultures in which ECs and PCCs are cultured independently (single culture), culturing ECs and PCCs together in 3D (co-culture) significantly enhances PCC growth, which is blocked by ruxolitinib (open bars). Data are mean ± SEM from three independent experiments. *P < 0.05, and **P < 0.01. **(C)** Schematic representation of PCC and EC cross-talk. TGF-β activates canonical Smad-dependent signaling in PCCs (top) leading to enhanced production of pro-angiogenic factors, which can be blocked by SB505124. These factors activate JAK/STAT3 signaling in ECs (bottom), which promotes EC proliferation through HDAC9, and ruxolitinib blocks these effects. ECs also produce factors (angiocrine factors) that can exert growth-stimulatory effects on PCCs through JAK/STAT3 signaling, which can also be targeted with ruxolitinib.

To determine if ruxolitinib exerts tumor suppressive effects in KRC mice by targeting the endothelium, the neoplastic epithelium, or both compartments, we next co-cultured fluorescently-labeled SVEC4–10 ECs and KRC PCCs in a 3-dimensional (3D) culture system [32]. Remarkably, PCC growth was enhanced in the co-culture model compared with 3D cultures in which PCCs were cultured separately from ECs, and this enhanced growth in co-culture was completely suppressed by ruxolitinib (Figure 6A). By contrast, ruxolitinib failed to inhibit the growth of either PCCs or ECs when cultured separately (Figure 6B). Thus, ECs can enhance PCC growth through an angiocrine mechanism, which is suppressible by targeting JAK1–2 with ruxolitinib (Figure 6C).

## DISCUSSION

It is projected that PDAC will become the second leading cause of cancer death in the US by 2030 [47]. It is crucial, therefore, to develop a personalized therapeutic approach for PDAC. However, this is a daunting task given the complexity of the molecular alterations and mutations in PDAC. Moreover, only ~20% of patients undergo resection, and tissue biopsies obtained from the remaining PDAC patients (~80%) are generally too small for molecular analysis such as deep sequencing or RNASeq, in part due to the clinical imperative to analyze the samples in a manner that assures adequate patient care. Importantly, these samples consist of a mixed population of cells with extensive stroma, resulting in a paucity of cancer cells within the biopsy sample. By contrast, fragments from surgically resected samples can be implanted subcutaneously in immune-deficient mice to yield patient-derived xenografts (PDX), allowing the cancer cells to grow in vivo in sufficient quantities for investigators to conduct extensive cancer genome analyses [48]. Here we show that directly implanting EUS-FNA-derived samples into the pancreas of immune-deficient mice yields viable human orthotopic tumors termed PDOX.

Tumor angiogenesis is generally not believed to play a role in PDAC pathobiology. However, our transcriptome analysis of TCGA data served to identify a subset of PDACs exhibiting a strong angiogenic gene signature, and we used the PDOX model to confirm that angiogenesis occurs in PDAC. Thus, we determined that in their earliest *in vivo* passage (F0), PDOX tumors harbor many human ECs, pointing to their presence in the original tumors and underscoring their ability to survive for many months in the tumor microenvironment in this model. The intra-pancreatic location of the PDOX tumors avoids the issues that may occur with subcutaneous PDX models where blood flow derives from the subcutaneous compartment which is not relevant to PDAC. Importantly, PDOX tumors can be established from all PDAC patients, including the 80% of patients who are ineligible for surgical resection, and they can be established using relatively small tissue biopsies to generate tumors that recapitulate histological features of the original patient tumor, including moderate or poorly differentiated tumors with varying degrees of desmoplasia. Thus, our PDOX model could be useful for testing drug responses and designing strategies for personalized medicine.

mPDACs arising in KIC mice which express oncogenic Kras and are devoid of the Ink4a locus encoding p16 exhibit angiogenesis histologically and express pro-angiogenic factors [49]. In the present study we determined that KRC mice, which harbor oncogenic Kras and are devoid of RB [27], also exhibited tumor angiogenesis and a pro-angiogenic gene signature that partly overlapped with genes expressed at high levels in the angiogenic subset of TCGA PDACs, as further confirmed by GSEA. Moreover, intravital microscopy established the presence of blood flow in the KRC tumors, similar to observations in animal models of vascular human cancers [50, 51]. By contrast, KPC mice develop hypovascular mPDACs that have minimal blood flow [17, 46, 52], KTC mice (oncogenic Kras with deletion of the type II TGF-β receptor) exhibit tumor angiogenesis [33], whereas in KPfl/+CY mice (oncogenic Kras and heterozygous p53 loss) exhibit suppressed angiogenesis [20]. Although targeting the stroma in KTC and KPfl/+CY mice promotes undifferentiated mPDAC, our current analysis of human PDACs did not demonstrate a clear correlation between tumor angiogenesis and either stroma abundance or tumor differentiation. Taken together, these observations point to marked differences in tumor angiogenesis among different GEMMs of PDAC, and suggest that our PDOX model accurately reflects the status of tumor angiogenesis in PDAC patients. Moreover, given that KRAS and p16 are the two most commonly mutated genes in PDAC, and that RB dysfunction is also common in PDAC [32], KRC mice could be a useful GEMM for studying angiogenesis that reflects events in PDAC patients whose cancers exhibit a pro-angiogenic signature.

In assessing the mechanisms that promote angiogenesis in the KRC GEMM, we determined that CM from KRC PCCs enhanced EC growth and migration, and activated canonical TGF-β and STAT3 pathways in cultured SVEC4–10 ECs. Although TGF-β and STAT3 are known to enhance tumor angiogenesis [36, 53, 54], TβRI inhibition with SB505124 did not suppress endothelial activation *in vitro*. Instead, it decreased the expression of several pro-angiogenic factors in KRC PCCs and suppressed mPDAC angiogenesis *in vivo*. Thus, the pro-angiogenic actions of TGF-βs in KRC mPDAC are not due to a direct effect by TGF-β on ECs. By contrast, JAK1–2 inhibition with ruxolitinib or STAT3 silencing prevented PCC-derived factors from enhancing STAT3-dependent transcription, as determined in a STAT3 luciferase reporter assay. Moreover, STAT3 silencing attenuated HDAC9 expression, and prevented CM from up-regulating HDAC9 in ECs. HDAC9 is a member of the class II family of histone deacetylases that includes HDACs 6 and 7, and all three HDACs exert pro-angiogenic effects in ECs [55–57]. Whereas HDAC6 and 7 were not part of the pro-angiogenic signatures in human PDACs or KRC mPDACs, HDAC9 was part of both signatures, was abundant within ECs in human PDAC, and in PDOX and KRC tumors, and enabled PCC-derived factors to stimulate proliferation of ECs that lack STAT3. Thus, HDAC9 promotes angiogenesis downstream of STAT3 and could serve as a marker of angiogenesis and endothelial STAT3 activation in PDAC.

Our findings thus point to a model in which TGF-βs promote the expression of pro-angiogenic factors in PCCs that, in turn, activate ECs through STAT3-dependent pathways (Figure 6C). Two distinct observations support this conclusion. First, the pro-angiogenic signature in KRC mPDACs correlated with an active TGF-β gene signature, as determined by GSEA. Second, TGF-β increased the levels of STAT3-activating pro-angiogenic cytokines in KRC-derived CM, including VEGF-A and GM-CSF. Oncogenic Kras has been previously shown to upregulate VEGF-A [58] and GM-CSF expression [59]. Importantly, in addition to enhancing angiogenesis, GM-CSF can promote mPDAC progression by recruiting myeloid-derived suppressor cells (MDSCs) which antagonize cancer-directed immune mechanisms [59, 60]. Thus, suppressing TGF-β pathways in PDAC can lead to attenuated angiogenesis and enhanced cancer directed immune activity by attenuating GM-CSF expression and by suppressing the direct actions of TGF-β on immune cells.

EC-derived factors are known to promote the proliferation of epithelial-type cells through paracrine interactions during hepatic cell regeneration [61], and in lymphoma [62], ovarian cancer [63], and breast cancer [64]. Using a 3D co-culture system we demonstrated for the first time that activated ECs promote PCC proliferation, and that this action is mediated by STAT3-dependent pathways. STAT3 is a known oncogene and survival factor that promotes cancer cell proliferation, tumor angiogenesis, and inflammation [53, 54, 65]. Moreover, when oncogenic Kras is combined with loss of p53, both EGFR and STAT3 must be suppressed to block mPDAC development [66], underscoring the importance of STAT3 in the earliest stages of PDAC progression. STAT3 is activated by JAK1–2, and the JAK1–2 inhibitor ruxolitinib has been approved by the FDA for the treatment of myelofibrosis and polycythemia vera, and is currently being tested in pancreatic and breast cancer clinical trials. In addition to modulating PCC-autonomous actions, and cancer-associated inflammatory signaling, our findings suggest that STAT3 acts in ECs to promote tumor angiogenesis, and facilitate angiocrine actions. Thus, it was important to assess the consequences of ruxolitinib administration in the KRC GEMM. Impressively, when administered at an age when mPDAC has already developed, ruxolitinib suppressed tumor angiogenesis and disease progression in KRC mice, dramatically prolonging their survival. By contrast, ruxolitinib did not have any beneficial effects in KPC mice, which are hypovascular and did not exhibit a pro-angiogenic gene signature.

The current findings suggest that targeting JAK1–2 with ruxolitinib could attenuate the proliferation of PCCs and associated ECs while dampening the actions PDAC-associated inflammatory cells and inflammatory cytokines, especially in those patients whose cancers express a strong pro-angiogenic signature. Taken together with recent encouraging Phase II data in metastatic PDAC using vatalanib, which targets pro-angiogenic VEGFRs and PDGFRs [26], and ruxolitinib [67], our TCGA analysis and the KRC GEMM data raise the possibility that PDAC patients should be selected for anti-angiogenesis therapy based on their pro-angiogenic gene signature. Our findings also suggest that ruxolitinib may be especially effective because it targets the JAK-STAT3 pathway that is downstream of multiple pro-angiogenic factors, and suggest that selection of patients for this type of therapeutic targeting may also exert beneficial actions by interrupting growth-promoting angiocrine pathways.

## METHODS

### Cell lines and conditioned media

KRC cells were cultured as described [32]. SVEC4–10 ECs were obtained from ATCC (CRL-2181). PANC-1 (CRL-1469) pancreatic cancer cells were established 39 years ago [68], and Panc 08.13 (CRL-2551) were established more recently [69]. Both are from primary pancreatic adenocarcinomas [68], have *KRAS^G12D^* mutations [69, 70], and were obtained from ATCC. ECs and human PCCs were cultured in DMEM supplemented with 1% antibiotic (100 units/ml penicillin; 100 mg/ml streptomycin), and FBS (10%/ECs; 5%PCCs). KRC tumor ECs were established from 2 month-old KRC pancreata as described [49]. All cell lines were confirmed to be mycoplasma-free using the Mycoalert detection kit (Lonza). For conditioned media (CM), KRC cells were seeded in 10 cm tissue culture plates (BD Falcon). 72 h post-serum starvation, media was collected, centrifuged and filtered through a 0.22 μm syringe filter.

### Mice

KRC and KPC mice were generated and maintained as described [16, 32]. For intravital imaging, Tetramethylrhodamine (TRITC)-conjugated Dextran (80 mg/kg) and Hoescht (10 mg/kg) were prepared in sterile saline, and injected into the tail vein of anesthetized KRC mice. A lateral incision was made, and the pancreas was exposed and imaged using a Leica TCS SP8 confocal microscope. Mice remained under anesthesia for the entire experiment. For therapeutic studies, 8 KRC mice or 8 KPC mice were randomized into ruxolitinib (4 mice) or vehicle control (4 mice) groups at postnatal weeks 4 or 12, respectively. For survival studies, 7 KRC mice were randomized into ruxolitinib (4 mice) or vehicle control (3 mice) groups. Ruxolitinib [50 mg/kg] or equal volumes of vehicle (0.5% hydroxypropyl methylcellulose (HPMC)) were administered to mice by daily gavage. All mice were genotyped twice (pre-weaning and post-necropsy).

To establish EUS-PDOX tumors, all patients signed informed consent and Dr. Cote and Dr. Sherman performed EUS-FNA procedures and placed them into DMEM/F12 supplemented with 2% antibiotic-antimycotic solution (Life Technologies). Two tissue fragments (1mm^3^ each) were washed 5x in media containing antibiotic-antimycotic, then implanted into the pancreata of 8 week old, male athymic mice (Harlan Laboratories). Human PCC orthotopic models were established by injecting 500,000 PANC-1 or Panc 08.13 cells into the pancreata of 8 week old athymic mice. All mouse studies were approved by the Institutional Care and Use Committee of Indiana University.

### Immunostaining

Pancreata from KRC mice were harvested at the indicated time points, and EUS-PDOX tumors were harvested as mice became moribund. Immunohistochemistry was performed as described [32]. Details, including antibodies and dilutions used are provided in the Supplementary Methods. For quantification, images were obtained at 20x magnification from 5 different fields using 3 mice using an Olympus BX60 microscope and a QImaging ExiBlue camera. Overall positive pixel intensity was determined using ImageProPlus v7.0 (Media Cybernetics). Quantitation data are presented as mean pixel intensity ± SEM. The human PDAC TMA was obtained from the Tissue Procurement and Distribution core at the Indiana University Simon Cancer Center. Scoring for stromal content was performed as described [10, 49, 71]. Briefly, tumors were scored independently by two investigators (J.G. and M.K.) for percentage of stromal content: + = 0–33%, ++ 33–67% and +++ = > 67%. Quantitation of immunohistochemistry using the TMA was performed using positive pixel count in Aperio Imagescope software. Approval for acquisition of the human PDAC TMA was granted by the Office of Research Administration at Indiana University.

### ELISA

KRC cells were seeded in 6-well plates (200,000/well). After 24 h, cells were serum-starved overnight, and treated with control media or TGF-β1 [0.5 nM] for 24 h. CM was prepared, and levels of the indicated cytokines were determined using a Milliplex ELISA kit (Millipore) per the manufacturer's recommendation.

### Microarrays

Array analysis of total RNA from KC and KRC cells was previously described [32]. Array analysis of KRC and KPC tumors was performed by Miltenyi Biotec. Briefly, Agilent whole mouse genome microarrays were performed using total RNA from 4 month-old KRC or 3 month-old KPC tumors (Cy5-labeled), or age- and sex-matched normal pancreata (Cy3-labeled). Details are provided in the Supplementary Methods. Data will be made available in the GEO database.

### TCGA analysis

RNASeq RSEM [72] normalized reads and raw count reads from the pancreatic ductal adenocarcinoma dataset (abbreviated PAAD) was downloaded from The Cancer Genome Atlas (TCGA) from http://cancergenome.nih.gov/. Details are provided in the Supplementary Methods.

### Quantitative PCR

Quantitative PCR (qPCR) was performed for the indicated mRNAs using Taqman gene expression assays (Life Technologies) and cDNA prepared from total RNA as described [27, 32]. *β-actin* and *Rps6* served as the endogenous controls for cells and tumor tissues, respectively.

### Cell proliferation

Cell proliferation was assessed by MTT [73]. Briefly, ECs (5,000/well) were seeded in 96-well plates, serum-starved, and media was replaced with serum-free media (control) or CM from KRC cells. For inhibitor studies, SB505124 [2 μM], ruxolitinib [100 nM] or DMSO [0.05%] were added to control and conditioned media. STAT3 knockdown was performed as described [74] by transducing ECs with two shRNAs that target murine STAT3 [75] or a non-targeting shRNA control (Thermo Scientific). An HDAC9 cDNA construct (Origene) was transfected into ECs using Lipofectamine 2000 (Life Technologies) per manufacturer's recommendations. An empty vector (pCMV6) was transfected as a control. Proliferation was assessed at 48 h. The mean of control-treated cells was normalized to 100%, and changes in proliferation were calculated as % control in three independent experiments.

### Migration

Migration was assessed using a Boyden chamber assay. ECs (20,000) were seeded in 8.0 μm cell culture inserts (BD Biosciences) in serum-free media, and placed into 24-well plates containing serum-free media, or CM. For inhibitors, SB505124 [2 μM], ruxolitinib [100 nM] or DMSO [0.05%] were added to the inserts and wells. After 16 h, cells were fixed in methanol and stained. The total number of cells that migrated was counted.

### Luciferase assays

ECs (25,000) were seeded in 24-well culture plates. After 24 h, ECs were transfected with a STAT3 luciferase reporter (Promega), and Renilla to control for transfection efficiency, using Lipofectamine 2000. After overnight serum starvation, media was replaced with control media or CM for 24 h, and luciferase assays were performed on three independent experiments as described [32].

### 3-dimensional culture

KRC and SVEC4–10 cells were cultured in 3D as described [32]. Briefly, KRC PCCs were transduced with an eGFP lentiviral construct (Clontech), and SVEC4–10 ECs were labeled using the PKH26 red fluorescent cell linker kit per the manufacturer's recommendations (Sigma-Aldrich). 3,000 PCCs or 6,000 ECs were cultured alone or together in 3% matrigel. Two days after plating, and every two days thereafter, cells were treated with control media, or media with ruxolitinib [100 nM]. The final concentration of DMSO in all experiments was 0.05%. Images were acquired with a Leica DMI3000 inverted microscope outfitted with a DFC300 FX camera. Fluorescence intensity was determined on three independent experiments plated in duplicate using Image Pro Plus v.7 (Media Cybernetics).

### Immunoblotting

ECs (200,000/well) were seeded in 6-well plates, serum-starved, and media was replaced with control or conditioned media containing SB505124 [2 μM], ruxolitinib [100 nM] or DMSO [0.05%]. Lysates were prepared and immunoblotting was performed as described [32]. For STAT3 knockdown, lysates were prepared from control- and shRNA-transduced cells 48h post-seeding.

### Statistical analysis

One-way ANOVA with Tukey's post-hoc test, one-tailed Student's *t*-test, or log rank Kaplan-Meier survival analysis was used to test for significant differences using Sigma Plot v.11.0 software (Systat Software). All statistics were performed on triplicate experiments. A *P* < .05 was considered statistically significant, and asterisks denote significant differences.

## SUPPLEMENTARY METHODS


