# Supplementary material for: TCGA data and patient-derived orthotopic xenografts highlight pancreatic cancer-associated angiogenesis
Source: Oncotarget. 2015 Feb 25;6(10):7504–21. doi: 10.18632/oncotarget.3233 (PMC4480696; doi:10.18632/oncotarget.3233)
Supplement: Supplementary file 1 [file oncotarget-06-7504-s001.pdf]

SUPPLEMENTARY METHODS

Immunohistochemistry

Tissues were harvested, fixed in 10% formalin overnight and embedded in paraffin. 4 µm sections were prepared using a HM355S microtome (Thermo Scientific). After deparaffinization and tissue rehydration, antigen retrieval was performed with antigen unmasking solution (Vector Labs, Burlingame, CA) or EDTA. Immunohistochemical detection was using biotinylated secondary antibodies, and a NOVA RED detection kit (Vector Labs). Masson's Trichrome (Richard-Allan Scientific) and Picosirius Red (Polysciences, Inc.) were performed according to manufacturer's recommendations.

Microarrays

Murine array intensity values were extracted, converted to log2-scale, and LOESS normalization was performed. Unpaired t-tests with equal variance were used to test log2-normalized data for significant differences. *P*-values were subjected to multiple testing (Benjamini-Hochberg) correction to reduce false discovery rate (FDR). Differentially expressed genes were considered statistically significant using fold change (FC: -1.5-fold and 1.5-fold), *P*-value ($P < .001$) and FDR (FDR < 0.05) cutoffs.

For gene ontology (GO) analysis, each probeset on the array (55,681 probesets), gene annotation information, including the Entrez Gene ID, RefSeq ID or gene symbol if available, were used to identify the associated Mouse Genome Informatics (MGI) ID. The final MGI ID gene list and the background gene list were uploaded on the Database for Annotation, Visualization and Integrated Discovery site (DAVID, version 6.7), and an analysis was run using the MGI ID as the identifier. The enriched GO terms in the biological process FAT ontology were focused on, and the visualization tool in AmiGO was used to generate the graphical GO graphs. For ingenuity pathway analysis (IPA), Agilent probe IDs, fold changes and *P*-values were uploaded into IPA, and a core analysis was performed using FC (-1.5-fold and 1.5-fold) and *P*-value ($P < .01$) cutoffs.

TCGA analysis

From the PAAD TCGA data, 580 Human UniProt IDs directly or indirectly annotated to the gene ontology [1] term angiogenesis (GO:0001525) were mapped to

List of Antibodies Name	Vendor	Dilution	Application
CD31	Abcam	1:200	IHC
Anti-human CD34	Abcam	1:200	IHC
Anti-human VE-Cadherin	BD Biosciences	1:200	IHC
CD105 (Endoglin)	Biolegend	1:200	IHC
Anti-mouse CD34	Millipore	1:50	IHC
Anti-mouse VE-Cadherin	BD Biosciences	1:50	IB
p-Smad3	Millipore	1:1000	IB
CK19	DSHB	1:10	IHC
p-STAT3 (Y705)	Cell Signaling	1:200/1:2000	IHC/IB
p-Histone H3 (S10)	Cell Signaling	1:200	IHC
STAT3	Cell Signaling	1:1000	IB
p-Smad2	Cell Signaling	1:1000	IB
p-Smad3	Millipore	1:1000	IB
Smad2/3	Cell Signaling	1:1000	IB
HDAC9	Origene	1:150	IHC

IHC = Immunohistochemistry, IB = Immunoblot

389 Entrez gene ids via use of the BioMart Bioconductor package [2–5], and 384 had expression values in the TCGA dataset. Hierarchical clustering was performed in R by applying a Pearson correlation distance and average linkage function to the normalized RSEM values of the 384 genes for the 85 tumor samples, and then scaling and graphing the result using the heatmap.2 function of the gplots R package. Because the resulting dendrograms indicated there was a subset of patients with up-regulated angiogenesis genes, we zoomed in on a dendrogram leaf of 129 genes, and then reclustered the data as follows. The normalized RSEM values for the 129 genes for the 85 tumor samples were first centered and scaled in R, and then hierarchical clustering on the rows was done using the Pearson correlation distance and average linkage function while column clustering was done using the Euclidian distance and complete linkage function.

Differential expression analysis between the strong and weak groups was carried out using DESeq (7) on the raw count data with 77 significantly changed genes meeting the following cutoff: FC ≥ 1.5; $P < 0.01$; FDR < 0.05. For comparison to KRC tumors, the 129 Entrez gene IDs were mapped to 127 Mouse Genome Informatics (MGI) ids by using the Vertebrate Homology file available at MGI. Of the 77 differentially expressed genes in the human dataset, 73 had mouse homologs, and 37 of those were also differentially expressed in the same direction according to the same cutoffs.

Gene set enrichment analysis (GSEA)

For KRC tumors, normalized, log2-transformed data from the were prepared in GSEA format [6]. A custom chip file that mapped probesets from this array to HUGO gene symbols was generated. Using version 2.1.0 of the command line jar application, this dataset was then compared against the 77 TCGA strong angiogenesis gene signature (Figure 3E) and a TGF-β gene set [7] (Figure 5B) from the Molecular Signature Database (MSigDB), version 4.0. For the TCGA data, modified log2 fold changes generated from the strong vs. weak DESeq analysis were used to rank the genes.

REFERENCES

1. Ashburner M, Ball CA, Blake JA, Botstein D, Butler H, Cherry JM, Davis AP, Dolinski K, Dwight SS, Eppig JT, Harris MA, Hill DP, Issel-Tarver L, Kasarskis A, Lewis S, Matese JC, et al. Gene Ontology: tool for the unification of biology. Nat Genet. 2000; 25:25–29.
2. Durinck S, Moreau Y, Kasprzyk A, Davis S, De Moor B, Brazma A, Huber W. BioMart and Bioconductor: a powerful link between biological data-bases and microarray data analysis. Bioinformatics. 2005; 21:3439–3440.
3. Durinck S, Spellman PT, Birney E, Huber W. Mapping identifiers for the integration of genomic datasets with the R/Bioconductor package biomaRt. Nat Protocols. 2009; 4:1184–1191.
4. Gentleman R, Carey V, Bates D, Bolstad B, Dettling M, Dudoit S, Ellis B, Gautier L, Ge Y, Gentry J, Hornik K, Hothorn T, Huber W, Iacus S, Irizarry R, Leisch F, et al. Bioconductor: open software development for computational biology and bioinformatics. Genome Biology. 2004; 5:R80.
5. Reimers M, Carey VJ. Bioconductor: Anpen Source Framework for Bioinformatics and Computational Biology. Methods in Enzymology. Alan K, Brian O. Methods in Enzymology. Academic Press2006; :119–134.
6. Subramanian A, Tamayo P, Mootha VK, Mukherjee S, Ebert BL, Gillette MA, Paulovich A, Pomeroy SL, Golub TR, Lander ES, Mesirov JP. Gene set enrichment analysis: a knowledge-based approach for interpreting genome-wide expression profiles. Proceedings of the National Academy of Sciences. 2005; 102:15545–15550.
7. Labbé E, Lock L, Letamendia A, Gorska AE, Gryfe R, Gallinger S, Moses HL, Attisano L. Transcriptional Cooperation between the Transforming Growth Factor-β and Wnt Pathways in Mammary and Intestinal Tumorigenesis. Cancer Research. 2007; 67:75–84.

SUPPLEMENTARY FIGURES AND TABLES

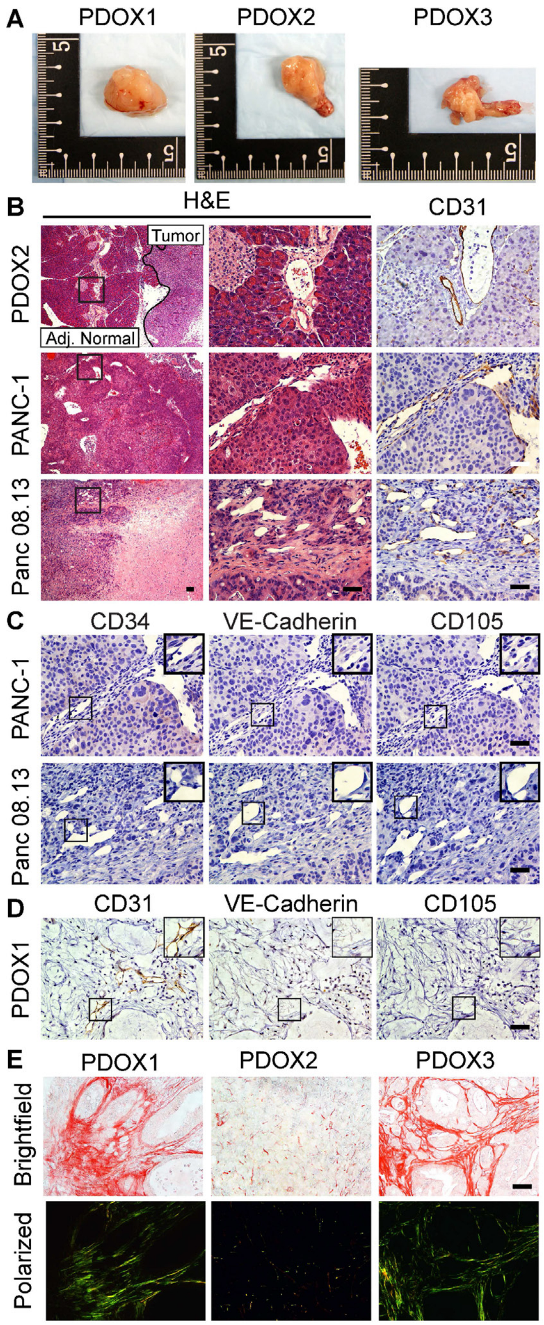

Supplementary Figure 1: EUS-FNA tissue samples form tumors in athymic mice. (A) Implantation of EUS-FNAs into pancreata of athymic mice yields large, intrapancreatic tumors. Shown are representative images from 3/6 mice. Scale, cm. **(B)** A CD31 antibody reacts with murine ECs as evidenced by the presence of immunoreactivity in the normal murine pancreas adjacent (Adj. normal) to EUS-PDOX tumors (PDOX2, top), and in tumors generated by intrapancreatic injection of PANC-1 (middle) or Panc 08.13 (bottom) PCCs. Shown are low (left) and high (middle) magnification images of H&Es, and CD31 immunostaining (right). **(C)** Human-specific CD34, VE-Cadherin and CD105 antibodies to not react with ECs in tumors generated by intrapancreatic injection of PANC-1 (middle) or Panc 08.13 (bottom) PCCs. **(D)** In their first in vivo passage (F1), EUS-PDOX tumors harbor CD31-positive endothelial cells (left), but these are host-derived as evidenced by the absence of immunoreactivity using human-specific VE-cadherin (middle) or CD105 (right) antibodies. **(E)** PDOX1 (left) and PDOX3 (right) exhibit an abundant stroma, rich in collagens as evidenced by Picosirius Red staining (top) and polarized light microscopy (bottom). Insets in (C–D) show magnified images of boxed areas. Scale bars in (B–E), 50 µm.

A

Resectable Tumors

	**Patient tumors**				**F1 EUS-PDOX tumors**		
**ID**	**Diagnosis**	**Stage**	**Differentiation**	**Size (cm)**	**Differentiation**	**Stroma**	**MVD**
PDOX1	Mucinous Adenocarcinoma	T3N1Mx	Moderate	3.7	Moderate	2	2
PDOX5	Adenocarcinoma	T3N1Mx	Moderate	3.4	Moderate	2	2

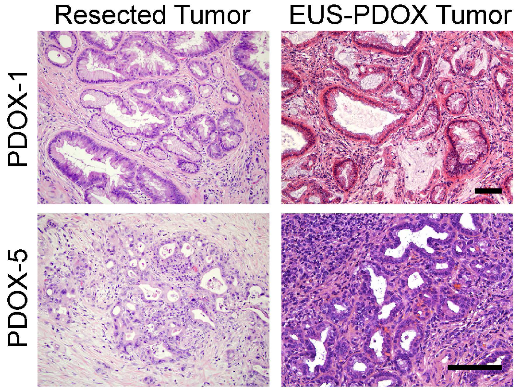

B

Un-resectable Tumors

	**Patient tumors**				**F1 EUS-PDOX tumors**		
**ID**	**Diagnosis**	**Stage**	**Differentiation**	**Size (cm)**	**Differentiation**	**Stroma**	**MVD**
PDOX2	Poorly Differentiated Carcinoma	T4NxMx	Poor	5.4	Poor	1	1
PDOX3	Adenocarcinoma	T4NxM1	N/A	3.8	Moderate	1	2
PDOX4	Adenocarcinoma	T4NxM1	N/A	4.7	Poor	2	1

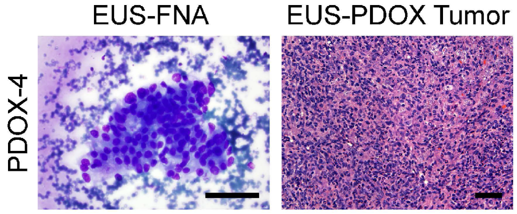

Supplementary Figure 2: Correlation of pathological findings in patients and F0 generation EUS-PDOX tumors. **(A)** The table (top) compares pathological features of surgically resectable patient tumors with their corresponding EUS-PDOX tumors. X: could not be determined. H&E staining (bottom) shows that surgically resected and F0 EUS-PDOX tumors exhibit similar histology. **(B)** The table (top) compares pathological features of un-resectable patient tumors with their corresponding EUS-PDOX tumors. N/A: not available. H&E staining shows the histology of EUS-FNA-4 and its corresponding EUS-PDOX tumor. H&Es of FNAs and tumors for PDOX2 and PDOX3 are shown in Figure 1. Scale bars, 50 μm.

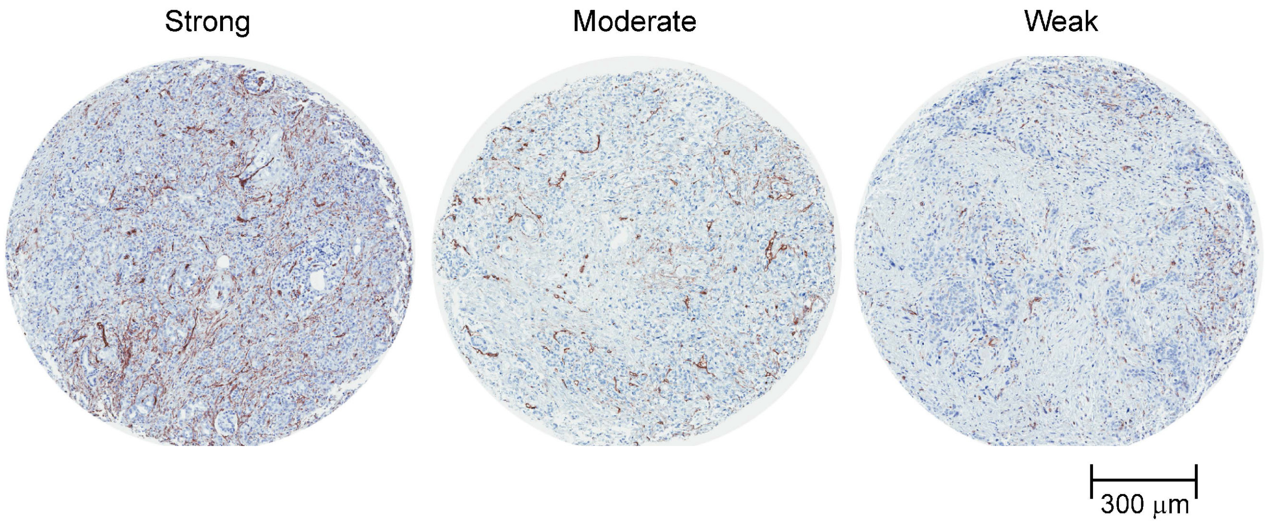

Supplementary Figure 3: A subset of PDACs exhibit robust angiogenesis. CD31 immunoreactivity is abundant and strong (left) in some PDAC tissues, but moderate (middle) or weak (right) in other PDACs. Shown are representative images acquired by Aperio slide scanning.

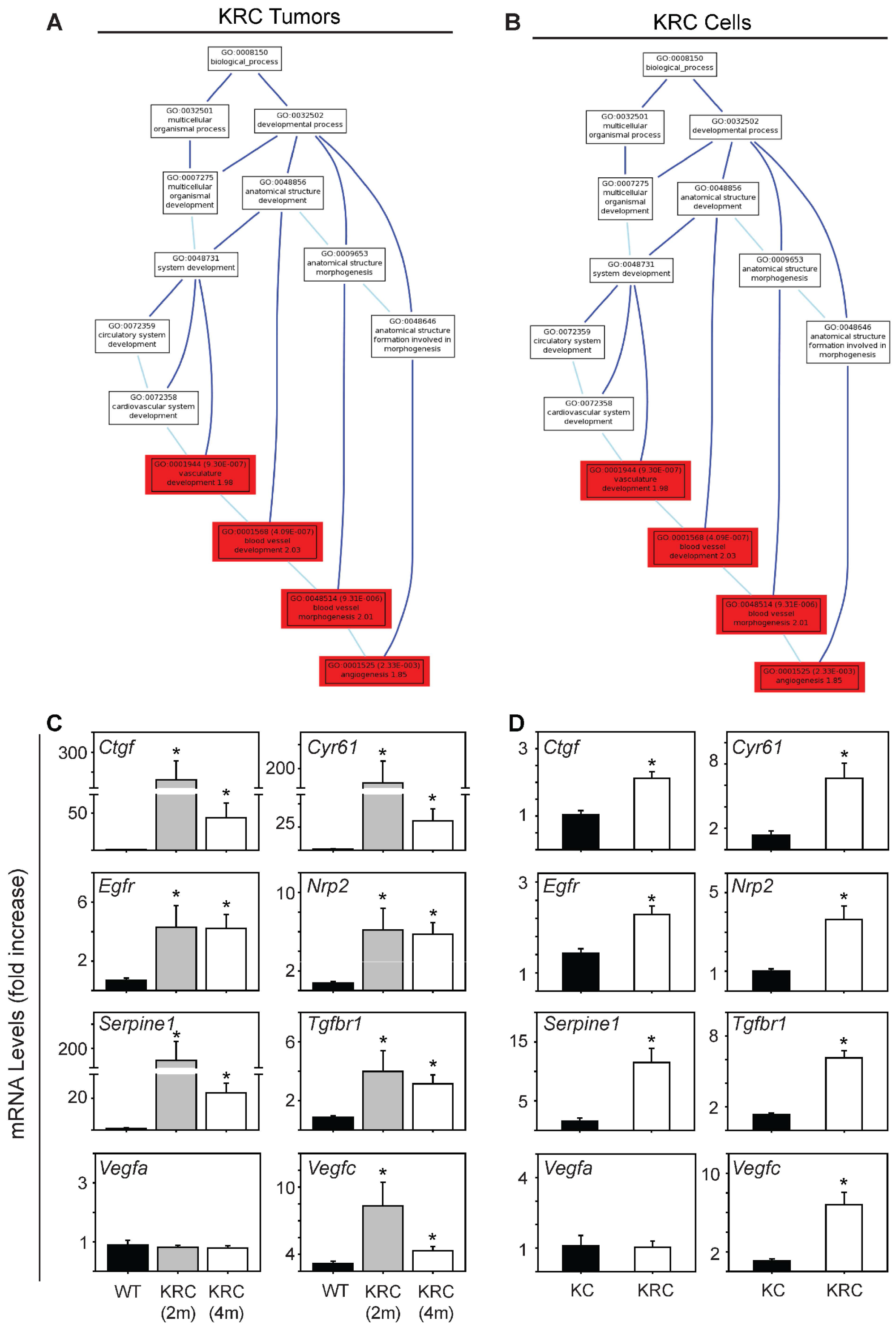

Supplementary Figure 4: Genes annotated to angiogenic processes are up-regulated in KRC tumors and cells. (A–B) Gene ontology (GO) analysis of differentially expressed genes in KRC tumors (A) and cells (B) shows that genes annotated to vasculature development, blood vessel development, blood vessel morphogenesis are significantly ($P < 0.01$) enriched. **(C–D)** Quantitative PCR (qPCR) for the indicated mRNAs validates the array data, and confirms that *Ctgf*, *Cyr61*, *Egfr*, *Nrp2*, *Serpine1*, *Tgfbr1* and *Vegfc* are significantly increased in 2 (gray bars) and 4 month-old (open bars) KRC tumors (C) compared with their respective littermate controls (WT, closed bars), and in KRC cells (D, open bars) compared with KC cells (closed bars). Data in (C–D) are mean ± SEM. *$P < 0.05$.

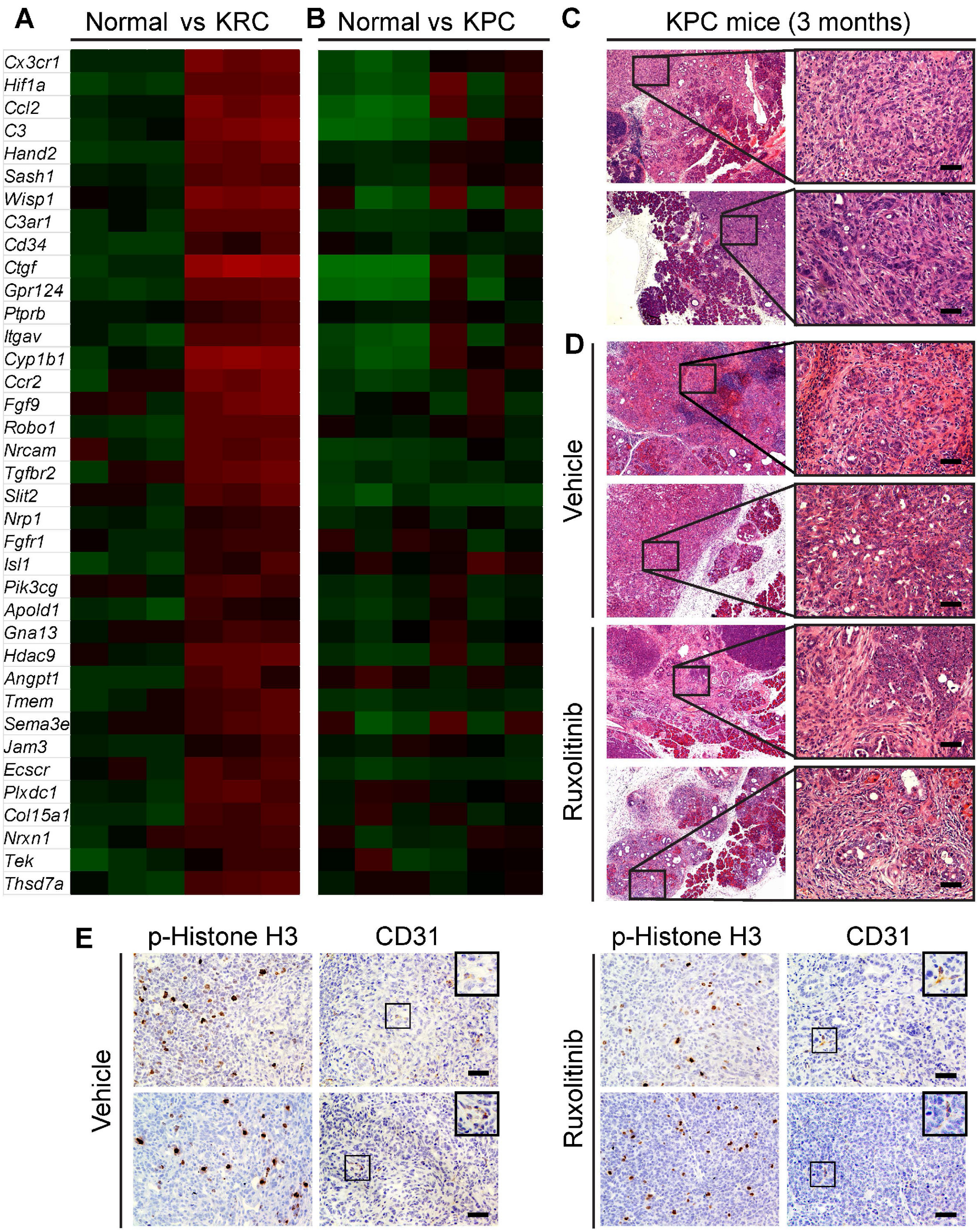

Supplementary Figure 5: KPC mice lack a tumor angiogenesis gene signature and do not benefit from ruxolitinib. **(A–B)** Heatmaps comparing array data from KRC tumors (A) or KPC tumors (B) show that the 37 pro-angiogenic genes that are significantly up-regulated in KRC tumors (A) and correlate with a pro-angiogenic gene signature in human PDACs are not up-regulated in KPC tumors. **(C–D)** H&E staining shows that pancreata from 3 month-old KPC mice often exhibit ADM, PanIN and mPDAC (C), and that pancreata from vehicle- and ruxolitinib-treated KPC mice are similar, and exhibit an abundance of lesions and mPDAC (D). Right panels in (C–D) are high magnification images of boxed areas. **(E)** mPDACs in vehicle- and ruxolitinib-treated mice harbor highly proliferative cancer cells, as evidenced by the presence of strong, nuclear p-Histone H3 immunoreactivity, but few ECs, as evidenced by the paucity of CD31 immunoreactivity. Shown in (C–E) are representative images from two mice per group. Scale bars, 50 μm.

Supplementary Table 1: Genes annotated to angiogenesis GO terms are differentially expressed in a subset of PDACs. Shown is the differential expression analysis of PDACs with strong or weak angiogenesis signatures as determined by TCGA analysis. 77 genes are significantly (FC ≥ 1.5; $P < 0.01$; FDR < 0.05) up-regulated in tumors with a strong signature, and of these, 63 are pro-angiogenic whereas 14 are anti-angiogenic. Genes are ranked by *P*-value and FDR.

Number	Gene Symbol	Fold Change	*P*-value	FDR
		**Pro-angiogenic**		
1	*CYP1B1*	11.89	1.32E−20	1.30E−16
2	*SLIT2*	16.50	1.27E−18	6.28E−15
3	*THSD7A*	11.04	1.22E−14	1.20E−11
4	*CALCRL*	7.90	2.10E−14	1.48E−11
5	*TEK*	7.69	3.70E−13	1.74E−10
6	*CCR2*	9.05	1.31E−12	5.27E−10
7	*TMEM100*	7.83	2.11E−11	4.69E−09
8	*S1PR1*	5.94	3.42E−11	7.10E−09
9	*PIK3CG*	7.13	7.61E−11	1.33E−08
10	*ANGPT1*	7.09	5.84E−10	6.74E−08
11	*KDR*	5.01	1.51E−09	1.53E−07
12	*APOLD1*	4.97	4.18E−09	3.53E−07
13	*SASH1*	4.79	4.86E−09	3.99E−07
14	*ROBO1*	4.06	1.14E−07	5.63E−06
15	*NRXN1*	14.26	1.73E−07	7.91E−06
16	*COL15A1*	3.96	1.92E−07	8.70E−06
17	*CXCL12*	6.37	2.24E−07	9.93E−06
18	*CX3CR1*	5.75	2.30E−07	1.01E−05
19	*C3*	2.88	5.81E−07	2.15E−05
20	*PTPRB*	3.61	6.35E−07	2.28E−05
21	*WISP1*	4.52	7.73E−07	2.66E−05
22	*C3AR1*	3.73	1.24E−06	3.91E−05
23	*HDAC9*	3.86	1.36E−06	4.20E−05
24	*CYSLTR2*	8.86	2.32E−06	6.44E−05
25	*NRCAM*	5.62	7.78E−06	1.77E−04
26	*GPR124*	3.25	8.12E−06	1.84E−04
27	*ENPEP*	3.33	1.03E−05	2.22E−04
28	*TAL1*	3.82	2.11E−05	3.91E−04
29	*CD34*	3.03	2.55E−05	4.56E−04
30	*FGFR1*	2.83	2.90E−05	5.07E−04
31	*GJA5*	3.18	3.67E−05	6.07E−04

(*Continued*)

Number	Gene Symbol	Fold Change	*P*-value	FDR
**Pro-angiogenic**				
32	*FLT1*	2.88	4.04E−05	6.53E−04
33	*NRP1*	3.04	4.06E−05	6.55E−04
34	*SCG2*	16.90	4.61E−05	7.23E−04
35	*CTGF*	3.80	1.04E−04	1.38E−03
36	*CLIC4*	2.80	1.20E−04	1.55E−03
37	*CMA1*	15.91	1.58E−04	1.93E−03
38	*STAB1*	2.40	1.69E−04	2.03E−03
39	*GPLD1*	8.42	2.18E−04	2.48E−03
40	*PDE3B*	3.55	2.30E−04	2.59E−03
41	*TIE1*	2.70	2.81E−04	3.04E−03
42	*ISL1*	4.59	3.95E−04	4.02E−03
43	*TGFBR2*	2.49	5.92E−04	5.51E−03
44	*PIK3CA*	2.56	6.14E−04	5.67E−03
45	*CCL2*	4.03	6.16E−04	5.68E−03
46	*EPAS1*	2.40	6.63E−04	6.01E−03
47	*FLT4*	2.48	7.49E−04	6.65E−03
48	*HIF1A*	2.46	7.94E−04	6.94E−03
49	*NRXN3*	4.81	1.05E−03	8.62E−03
50	*SEMA3E*	4.16	1.44E−03	1.11E−02
51	*MCAM*	2.24	1.65E−03	1.23E−02
52	*ECSCR*	2.54	1.66E−03	1.23E−02
53	*PTEN*	2.05	2.83E−03	1.86E−02
54	*FGF9*	3.53	3.24E−03	2.07E−02
55	*SIRT1*	2.23	3.25E−03	2.07E−02
56	*HAND2*	2.31	3.67E−03	2.26E−02
57	*ITGAV*	2.06	4.16E−03	2.48E−02
58	*JAM3*	2.21	4.83E−03	2.79E−02
59	*PLXDC1*	2.09	5.63E−03	3.15E−02
60	*ACVRL1*	2.03	7.22E−03	3.80E−02
61	*GNA13*	1.96	7.58E−03	3.94E−02
62	*ROCK1*	2.03	7.87E−03	4.06E−02
63	*DICER1*	1.98	9.29E−03	4.60E−02
**Anti−angiogenic**				
1	*SFRP1*	13.61	6.50E−07	2.33E−05
2	*RORA*	4.04	2.78E−07	1.18E−05
3	*MEOX2*	4.53	4.80E−07	1.85E−05

(*Continued*)

Number	Gene Symbol	Fold Change	*P*-value	FDR
**Anti−angiogenic**				
4	*BAI3*	11.06	1.02E−06	3.33E−05
5	*NPR1*	3.42	2.43E−05	4.39E−04
6	*COL4A3*	6.05	9.23E−05	1.25E−03
7	*MMRN2*	2.37	2.86E−04	3.08E−03
8	*APOH*	5.06	3.04E−04	3.24E−03
9	*ELK3*	2.62	4.12E−04	4.14E−03
10	*VASH1*	2.48	4.95E−04	4.77E−03
11	*ROBO4*	2.44	6.31E−04	5.80E−03
12	*ROCK2*	2.42	9.92E−04	8.28E−03
13	*THBS4*	3.26	4.19E−03	2.49E−02
14	*PTPRM*	2.03	7.82E−03	4.04E−02

Supplementary Table 2: Differentially expressed genes in KRC tumors correlate with a TCGA angiogenesis gene signature. Out of 77 genes differentially expressed in human PDACs with a strong angiogenesis signature, 42 are differentially expressed (FC ≥ 1.5; $P < 0.01$; FDR < 0.05) in KRC tumors compared with normal murine pancreata. Of these, 37 are pro-angiogenic, whereas 5 are anti-angiogenic. Genes are ranked by *P*-value and FDR, and asterisks denote genes with 2 probes on Agilent murine arrays.

Number	Human Gene Symbol	Murine Probe ID	Fold Change	*P*-value	FDR
**Pro-angiogenic**					
1	*CX3CR1**	A_55_P2007964	7.79	2.00E−06	6.04E−04
2	*HIF1A*	A_51_P387608	6.43	4.00E−06	7.81E−04
3	*CCL2*	A_51_P286737	9.67	8.29E−06	9.74E−04
4	*C3**	A_55_P2038525	9.42	1.30E−05	1.15E−03
5	*HAND2*	A_55_P2016237	6.39	1.30E−05	1.15E−03
6	*SASH1*	A_52_P649224	3.09	2.00E−05	1.40E−03
7	*WISP1*	A_51_P220343	14.45	2.33E−05	1.53E−03
8	*C3AR1*	A_51_P282557	3.87	2.40E−05	1.55E−03
	*C3**	A_51_P110301	16.59	5.00E−05	2.05E−03
9	*CD34*	A_51_P204740	2.87	6.70E−05	2.32E−03
10	*CTGF*	A_51_P157042	36.13	6.72E−05	2.33E−03
11	*GPR124*	A_51_P282523	5.37	7.00E−05	2.38E−03
12	*PTPRB*	A_51_P290931	1.71	1.04E−04	2.94E−03
13	*ITGAV**	A_51_P382484	4.09	1.07E−04	2.99E−03
14	*CYP1B1*	A_51_P255456	7.02	2.97E−04	5.00E−03
15	*CCR2*	A_51_P245989	8.07	3.63E−04	5.58E−03
16	*FGF9*	A_55_P2015994	5.29	5.01E−04	6.71E−03

(*Continued*)

Number	Human Gene Symbol	Murine Probe ID	Fold Change	*P*-value	FDR
**Pro-angiogenic**					
17	*ROBO1*	A_55_P1985070	3.98	5.54E−04	7.09E−03
18	*NRCAM*	A_55_P2003541	3.96	5.79E−04	7.28E−03
19	*TGFBR2**	A_51_P450573	4.28	5.93E−04	7.38E−03
	*ITGAV**	A_55_P2039771	3.62	5.98E−04	7.41E−03
20	*SLIT2*	A_55_P1958394	2.76	6.50E−04	7.77E−03
	*TGFBR2**	A_55_P2107288	2.68	1.04E−03	1.03E−02
21	*NRP1*	A_51_P469285	1.82	1.36E−03	1.20E−02
22	*FGFR1*	A_55_P2057777	1.75	1.50E−03	1.28E−02
23	*ISL1*	A_52_P337246	2.50	2.27E−03	1.67E−02
24	*PIK3CG*	A_55_P2126033	1.76	2.51E−03	1.79E−02
25	*APOLD1*	A_55_P2062108	2.33	2.53E−03	1.80E−02
26	*GNA13*	A_51_P286563	1.53	2.56E−03	1.81E−02
27	*HDAC9*	A_55_P1970755	2.99	3.24E−03	2.12E−02
28	*ANGPT1*	A_55_P2158227	2.83	3.34E−03	2.16E−02
29	*TMEM100*	A_52_P368306	2.38	3.44E−03	2.21E−02
	*CX3CR1**	A_52_P99810	2.33	3.89E−03	2.40E−02
30	*SEMA3E*	A_55_P2087414	1.96	3.89E−03	2.40E−02
31	*JAM3*	A_51_P358354	1.76	4.26E−03	2.56E−02
32	*ECSCR*	A_52_P1026777	1.88	4.90E−03	2.83E−02
33	*PLXDC1*	A_55_P2364738	2.59	5.08E−03	2.90E−02
34	*COL15A1*	A_55_P2060379	2.37	7.31E−03	3.76E−02
35	*NRXN1*	A_52_P121342	1.96	8.96E−03	4.36E−02
36	*TEK*	A_55_P2262136	2.44	9.75E−03	4.63E−02
37	*THSD7A*	A_55_P2161219	2.66	9.96E−03	4.70E−02
**Anti−angiogenic**					
1	*SFRP1*	A_66_P134428	17.22	5.00E−05	2.05E−03
2	*ELK3**	A_55_P1972927	2.84	2.75E−04	4.79E−03
3	*THBS4*	A_52_P401504	16.27	3.29E−04	5.26E−03
	*ELK3**	A_55_P2018994	2.56	4.76E−04	6.51E−03
4	*MMRN2*	A_51_P414396	1.55	4.21E−03	2.54E−02
5	*ROCK2**	A_51_P320444	1.43	7.77E−03	3.93E−02
	*ROCK2**	A_65_P19458	1.66	9.25E−03	4.46E−02

Supplementary Table 3: Genes annotated to angiogenesis GO terms are differentially expressed in KRC cells. Shown are significantly (FC > or < 1.5; *P* < 0.01; FDR < 0.05) altered genes in KRC cells compared with KC cells. Genes are ranked by fold change and *P*−value. Asterisks denote predicted TGF−β target genes.

Number	Gene Symbol	Fold Change	*P*−value	FDR
**Pro−angiogenic**				
1	*Serpine1**	34.46	1.53E−08	2.09E−05
2	*Ccbe1**	24.33	1.08E−07	3.25E−05
3	*Nrp2**	19.75	1.86E−07	4.95E−05
4	*Wisp1**	17.28	7.36E−06	2.03E−04
5	*Ccl2**	16.37	7.33E−07	7.39E−05
6	*Cav1**	14.11	4.24E−08	3.01E−05
7	*Hspb1**	14.04	2.23E−06	7.37E−05
8	*Cyr61**	11.63	1.63E−06	9.74E−05
9	*Cxcl12**	10.99	1.47E−07	4.81E−05
10	*Flt1**	9.42	8.07E−07	3.89E−05
11	*Ptprb**	9.33	3.87E−07	6.23E−05
12	*Jam3**	8.79	6.33E−06	1.86E−04
13	*Ecscr**	6.94	1.26E−06	8.67E−05
14	*Inhba*	6.88	8.82E−07	7.63E−05
15	*Ptgs2**	6.66	8.93E−07	7.63E−05
16	*Fn1**	6.49	2.47E−05	4.15E−04
17	*Adam8**	5.47	1.80E−06	1.02E−04
18	*Egfr*	4.63	2.57E−07	2.80E−04
19	*Vegfc**	4.54	1.51E−06	9.57E−04
20	*Pdgfa**	4.37	1.10E−06	1.25E−04
21	*Prkd1**	4.29	5.11E−05	6.16E−04
22	*Pdgfb**	3.95	3.94E−05	5.22E−04
23	*Ctgf**	3.94	5.48E−06	1.74E−04
24	*Pgf**	3.42	2.33E−05	3.79E−04
25	*Tgfbr1*	3.33	4.90E−06	1.66E−04
26	*Col4a1**	3.23	9.50E−06	1.87E−04
27	*Hspg2**	3.18	6.33E−06	1.86E−04
28	*Tspan12**	3.17	1.99E−05	3.50E−04
29	*Edn1**	3.08	1.34E−04	1.15E−03
30	*Tgfb2*	3.05	3.02E−06	1.31E−04
31	*Syk*	3	8.38E−06	2.19E−04
32	*Fgf18*	2.99	5.01E−05	6.08E−04

(*Continued*)

Number	Gene Symbol	Fold Change	*P*−value	FDR
		**Pro−angiogenic**		
33	*Junb*	2.76	2.47E−06	1.18E−04
34	*Egfl7*	2.44	2.03E−04	1.51E−03
35	*Tnfaip2*	2.39	2.48E−05	3.92E−04
36	*Col4a2**	2.23	8.66E−06	2.22E−04
37	*Adam15**	2.14	1.23E−06	8.66E−05
38	*Rhob*	2.1	4.74E−04	2.72E−03
39	*Fgfr2*	2.04	3.29E−04	2.09E−03
40	*Jag1**	2.01	1.73E−05	3.25E−04
41	*Cd40**	1.98	2.02E−05	3.52E−04
42	*Bdnf**	1.98	4.87E−05	5.98E−04
43	*Col1a1**	1.91	2.51E−04	2.58E−03
44	*Cd44*	1.7	5.52E−05	4.72E−03
45	*Itgb5**	1.64	4.30E−05	5.51E−04
46	*Tgfb1*	1.52	1.26E−04	1.10E−03
47	*Myc**	1.51	2.28E−05	3.75E−04
48	*Icam1**	−1.57	4.76E−05	5.89E−04
49	*Notch1*	−1.66	3.38E−04	2.36E−03
50	*Ang*	−2.05	2.28E−04	1.64E−03
51	*Hmox1*	−2.15	2.37E−06	1.15E−04
52	*Ereg*	−2.22	2.21E−06	1.11E−04
53	*Mmp13**	−2.57	4.60E−06	1.61E−04
54	*Fzd5*	−2.91	2.16E−04	1.34E−03
55	*Ramp1*	−2.96	5.15E−05	6.18E−04
56	*Cx3cl1*	−3.64	5.35E−08	3.29E−05
57	*Fgf1*	−4.49	9.46E−06	2.30E−04
58	*Mmp3**	−11.39	2.88E−06	1.27E−04
		**Anti−angiogenic**		
1	*Ahr**	7.54	1.21E−06	8.63E−05
2	*Sparc**	6.89	2.13E−07	1.19E−04
3	*Mmp9**	3.45	7.78E−03	3.57E−04
4	*Timp1*	1.61	9.00E−05	1.58E−03
5	*Pten*	−1.55	5.75E−05	2.17E−03
6	*Timp3*	−1.55	6.63E−04	3.46E−03
7	*Ang4**	−2.01	4.99E−04	3.90E−04
8	*Il1rn*	−5.95	2.28E−06	8.55E−04

Supplementary Table 4: Cytokine levels in KRC conditioned media. Shown are the mean concentrations [pg/ml] ± SD in conditioned media from two KRC cell lines.

Cytokine	Basal	+ TGF−β1 [0.5 nM]
CSF1	0	0
Cxcl1	2103 ± 1000	1611 ± 136
Cxcl5	745 ± 93	226 ± 5
Cxcl12	0	0
EGF	0	0
FGF−2	0	0
Follistatin	0	0
GM−CSF	227 ± 25	3331 ± 256
HGF	0	0
IL−2	0	0
IL-3	0	0
IL-5	0	0
IL-6	0	0
IL-7	0	0
IL-10	0	0
IL-12	0	0
IL-15	0	0
IL-17	0	0
Leptin	0	0
Mcp-1	1653 ± 147	1412 ± 483
Mip-1α	0	0
TNF-α	0	0
VEGF-A	72 ± 28	3024 ± 1056
VEGF-C	23 ± 3	86 ± 6
VEGF-D	0	0
